# An efficient analytical reduction of detailed nonlinear neuron models

**DOI:** 10.1038/s41467-019-13932-6

**Published:** 2020-01-15

**Authors:** Oren Amsalem, Guy Eyal, Noa Rogozinski, Michael Gevaert, Pramod Kumbhar, Felix Schürmann, Idan Segev

**Affiliations:** 1 Department of Neurobiology, Hebrew University of Jerusalem, 9190401 Jerusalem, Israel; 2 Blue Brain Project, École polytechnique fédérale de Lausanne (EPFL), Campus Biotech, 1202 Geneva, Switzerland; 3 Edmond and Lily Safra Center for Brain Sciences, Hebrew University of Jerusalem, 9190401 Jerusalem, Israel

**Keywords:** Cellular neuroscience, Biophysical models, Ion channels in the nervous system

## Abstract

Detailed conductance-based nonlinear neuron models consisting of thousands of synapses are key for understanding of the computational properties of single neurons and large neuronal networks, and for interpreting experimental results. Simulations of these models are computationally expensive, considerably curtailing their utility. *Neuron_Reduce* is a new analytical approach to reduce the morphological complexity and computational time of nonlinear neuron models. Synapses and active membrane channels are mapped to the reduced model preserving their transfer impedance to the soma; synapses with identical transfer impedance are merged into one NEURON process still retaining their individual activation times. *Neuron_Reduce* accelerates the simulations by 40–250 folds for a variety of cell types and realistic number (10,000–100,000) of synapses while closely replicating voltage dynamics and specific dendritic computations. The reduced neuron-models will enable realistic simulations of neural networks at unprecedented scale, including networks emerging from micro-connectomics efforts and biologically-inspired “deep networks”. *Neuron_Reduce* is publicly available and is straightforward to implement.

## Abstract (web summary)

Realistic simulations of neurons and neural networks are key for understanding neural computations. Here the authors describe Neuron_Reduce, an analytic approach to simplify neurons receiving thousands of synapses and accelerate their simulations by 40–250 folds, while preserving voltage dynamics and dendritic computations.

## Introduction

Compartmental models (CMs) were first employed by Wilfrid Rall^1^ to study the integrative properties of neurons. They enabled him to explore the impact of spatio-temporal activation of conductance-based dendritic synapses on the neuron’s output and the effect of the dendritic location of a synapse on the time course of the somatic excitatory postsynaptic potential^2^. By simulating electrically distributed neuron models, Rall demonstrated how the cable properties of dendrites explain the variety of somatic excitatory postsynaptic potential (EPSP) shapes that were recorded at the soma of α-motoneurons, thus negating the dominant explanation at that time that the differences in shapes of the somatic EPSPs in these cells result from differences in the kinetics of the respective synapses. This was an impressive example that faithful models of the neuron (as a distributed rather than a “point” electrical unit) are essential for the correct interpretation of experimental results. Since Rall’s 1964 and 1967 studies using CMs, EPSP “shape indices” measured at the soma are routinely used for estimating the electrotonic distance of dendritic synapses from the soma.

Over the years, detailed CMs of neurons have provided key insights into hundreds of experimental findings, both at the single-cell and the network levels. A notable example at the single-cell level is the explanation as to why the somatic Na^+^ action potential propagates backward in the soma-to-dendrites direction and (typically) not vice versa^3^. CMs have also pinpointed the conditions for the generation of local dendritic Ca^2+^ spikes^4–6^ and provided an explanation for the spatial restriction of the active spread of dendritic spikes from distal dendrites to the soma^7^ and see also refs. ^8–14^. Today, detailed CMs are even being used for simulating signal processing in human pyramidal neurons, including their large numbers of dendritic spines/synapses^15^.

At the network level, detailed CMs are utilized for such noteworthy projects as large-scale simulations of densely in silico reconstructed cortical circuits^16,17^ and the overarching goal of the Allen Institute to simulate large parts of the visual system of the mouse^18,19^. Because detailed compartmental modeling is increasingly becoming an essential tool for the understanding of diverse neuronal phenomena, major efforts have been invested in developing user-friendly computer software that implements detailed CMs, the best known of which are NEURON^20^, GENESIS^21^, NeuroConstruct^22^, PyNN^23^, and, recently, BioNet^24^, NTS^25^, NetPyNE^26^, and Geppetto^27^.

Modern personal computers can simulate tens of seconds of electrical activity of single neurons comprising thousands of nonlinear compartments and synapses. However, they handle poorly cases where many model configurations need to be evaluated such as in large-scale parameter fitting for single-neuron models^5,28^, or when the dendritic tree is morphologically and electrically highly intricate and consists of tens of thousands of dendritic synapses, as with the human cortical pyramidal neurons^15^. When the aim is to simulate a neuronal network consisting of hundreds of thousands of such neurons, only very powerful computers can cope. For example, the simulation of a cortical network consisting of 200,000 detailed neuron models on the BlueGene/Q supercomputer takes several hours to simulate 30 s of biological time^17^.

To overcome this obstacle, two approaches have been pursued. The first involves developing alternative, cheaper, and more efficient computing architectures (e.g., neuromorphic-based computers^29,30^). These have not yet reached the stage where they can simulate large-scale network models with neurons consisting of branched nonlinear dendrites having a realistic number of synapses. The other approach is to simplify neuron models while preserving their input/output relationship as faithfully as possible. Rall^31^ was the first to suggest a reduction scheme in his “equivalent cylinder” model, which showed that, for certain idealized passive dendritic trees, the whole tree could be collapsed into a single cylinder that was analytically identical to the detailed tree. The “equivalent cylinder” preserves the total dendritic membrane area, the electrotonic length of the dendrites, and, most importantly, the postsynaptic potential (amplitude and time course) at the soma for a dendritic synapse when mapped to its respective electrotonic location on the “equivalent cylinder”^32,33^. However, this method is not applicable for dendritic trees with large variability in their cable lengths (e.g., pyramidal neurons with a long apical tree and short basal trees), conductance-based synapses, or for dendrites with nonlinear membrane properties.

Over the years, several different reduction schemes have been proposed; for example, a recent work mapped all the synapses to a single compartment, taking the filtering effect of the dendrites into account^34^. Other methods reduce the detailed morphology to a simplified geometric model while preserving the total membrane area^35–37^ or the axial resistivity^38^; see also refs. ^12,39,40^. However, these methods have a variety of drawbacks; in particular, they are either “hand fitted” and thus lack a clear analytical underpinning or are complicated to implement, and in some cases, their computational advantage for realistic numbers (thousands) of synapses is not quantified. Most of these methods do not support dendrites with active conductances^35,38,39,41,42^ and they have not been tested on a broad range of neuron types. Importantly, none of the previous methods provided an easy-to-use open access implementation. Thus, today there is no simple, publicly available reduction method for neuron models that can be used by the extensive neuroscience and machine-learning communities.

To respond to this need, the present study provides an analytic method for reducing the complexity of detailed neuron models while faithfully preserving the essential input/output properties of these models. Neuron_Reduce is based on key theoretical insights from Rall’s cable theory, and its implementation for any neuron type is straightforward without requiring hand-tuning. Depending on the neuron modeled and the number of synapses, Neuron_Reduce accelerates the simulation run-time by a factor of up to 250 while preserving the identity of individual synapses and their respective dendrites. It also preserves specific membrane properties and dendritic nonlinearities, hence maintaining specific dendritic computations. Neuron_Reduce is easy to use, fully documented, and publicly available on GitHub (https://github.com/orena1/neuron_reduce).

## Results

### Mapping of a detailed neuron model to a multi-cylinder model

**Fig. 1:**
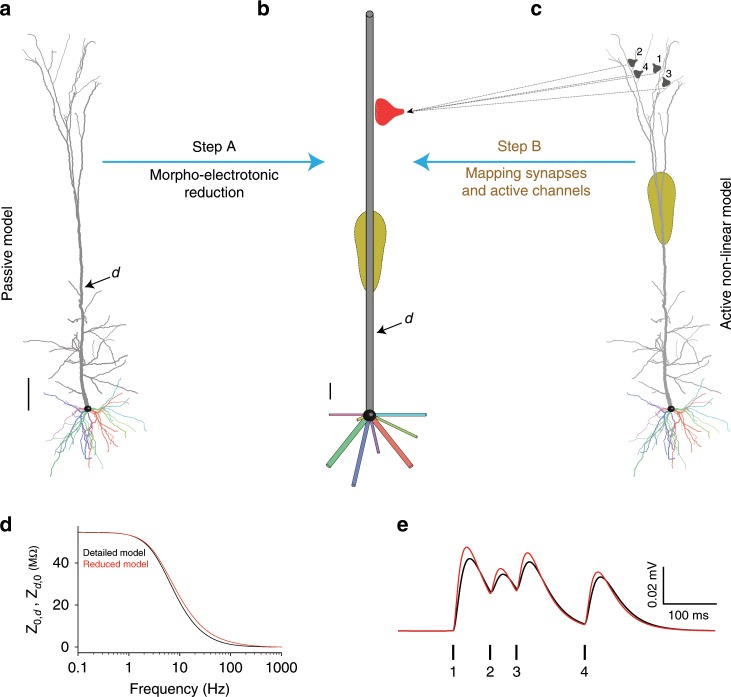
An analytic method for reducing neuron model complexity (Neuron_Reduce). **a** Detailed passive model of 3D reconstructed L5 thick-tufted pyramidal cell from rat neocortex. Its nine stem dendrites (one apical and 8 basal) are depicted in different colors. **b** Each original stem dendrite is reduced to a single cylinder that retains the specific passive cable properties (*R*_m_, *C*_m_, and *R*_a_) of the original tree. The diameter and length of the respective cylinders are computed analytically using Eqs. (1)–(11), such that each cylinder preserves both the transfer resistance from the most electrotonically distal dendritic tip to the soma as well as the input resistance at the soma end of the corresponding stem dendrite. This generates a unique cylindrical cable for each of the original stem dendrites. Scale bars in **a**, **b** are 100 µm. **c** Synapses with similar transfer resistance to the soma (exemplar synapses are marked as 1–4 at top right) are all mapped to the respective locus in the reduced cylinder so that their transfer resistance is similar in the two models. In the reduced model, these synapses are merged into one “NEURON” process (red synapse in **b**), but they retain their individual activation time (see Methods and Supplementary Fig. 1). The same mapping also holds for active membrane conductances (yellow region, denoting the Ca^2+^ “hot spot” in the apical tree). **d** Transfer impedance $(Z_{d,0} = Z_{0,d})$ between point *d* on the apical tree (shown in **a**, **b**) and the soma (*X* = 0) as a function of the input frequency in both the detailed (black trace) and the reduced (red trace) models. **e** Composite somatic EPSPs resulting from sequential activation of the four distal apical synapses shown in **c** in the detailed model (black trace) and the reduced model (red trace). In this simulation the dendritic tree was passive. The synapses were activated in temporal order 1, 2, 3, 4 as shown by the vertical lines below the composite EPSP. The respective peak conductances of these AMPA-based synapses were 0.6, 0.3, 0.4, and 0.4 nS (details in Supplementary Table 2 and see Supplementary Fig. 1 for the active case).

The thrust of our analytical reduction method (Neuron_Reduce) is described in Fig. 1a–c. This method is based on representing each of the original stem dendrites by a single cylindrical cable, which has the same specific membrane resistivity (*R*_m_, in Ωcm^2^), capacitance (*C*_m_, in F/cm^2^), and axial resistivity (*R*_a_, in Ωcm) as in the detailed tree (Fig. 1a). Also, each cylindrical cable satisfies two constraints: (i) the magnitude of the transfer impedance, $| {Z_{0,L}\left( \omega \right)} | = | {V_0\left( \omega \right)/I_L\left( \omega \right)} |$, from its distal sealed end (*X* = *L*) to its origin at the soma end (*X* = 0) is identical to the magnitude of the transfer impedance from the electrotonically most distal dendritic tip to the soma in the respective original dendrite; (ii) at its proximal end (*X* = 0), the magnitude of the input impedance, $| {Z_{0,0}\left( \omega \right)} | = | {V_0\left( \omega \right)/I_0\left( \omega \right)} |$, is identical to that of the respective stem dendrite (when decoupled from the soma). As shown in Eqs. (1)–(11) (Methods), these two constraints, while preserving the specific membrane and axial properties, guarantee a unique cylindrical cable (with a specific diameter and length) for each of the original dendrites.

Because the magnitude of the transfer impedance in both the original dendrite and in the respective cylindrical cable spans from $| {Z_{0,L}(\omega )} |$ to $| {Z_{0,0}(\omega )} |$, all dendritic loci having intermediate transfer impedance values can be mapped to a specific locus in the respective cylinder that preserves this intermediate transfer impedance. This mapping guarantees (for the passive case) that the magnitude of the somatic voltage response, *V*_0_(*ω*), to an input current, *I*_*x*_(*ω*), injected at a dendritic location, *x*, will be identical in both the detailed and the reduced cylinder models (see Methods). Consequently, synapses and nonlinear ion channels are mapped to their respective loci in the reduced cylinder while preserving the respective transfer impedance to the soma (see Fig. 1, Step B, and Methods). Based on Eqs. (1)–(11), Neuron_Reduce generates a reduced multi-cylindrical tree for any *ω* value (different reduced models for different *ω* values). Conveniently, we found a close match between the detailed and the reduced models for *ω* = 0 (the steady-state case). Therefore, all figures in this work are based on reduced models with *ω* = 0 (see Discussion).

### Neuron_Reduce implemented on L5 pyramidal cell with synapses

In Fig. 1, Neuron_Reduce is implemented on a detailed CM of a 3D reconstructed layer 5 pyramidal neuron from the rat somatosensory cortex (same model as in ref. ^5^). This neuron consists of eight basal dendrites and one apical dendrite (shown in different colors) stemming from the soma. This neuron model has active membrane ion channels at both the soma and dendrites (see below). However, Neuron_Reduce first treats the modeled tree as passive by abolishing all voltage-dependent membrane conductances, and only retaining the leak conductance. Implementing Eqs. (1)–(11) for this cell produced a reduced, multi-cylindrical, passive model (Fig. 1b, Step A) consisting of only 50 compartments rather than the 642 compartments in the detailed model.

Figure 1c shows an example of four synapses located at different apical branches. These synapses all have the same transfer resistance to the soma in the detailed tree. Therefore, Neuron_Reduce maps these synapses to a single respective locus in the respective cylinder, such that their transfer resistance is identical in both models. In the reduced model, these synapses are merged into one “NEURON” process (red synapse in Fig. 1b). However, they retain their individual activation times (see Methods). Figure 1d compares the transfer impedance between a specific point in the apical tree (marked by “*d*” in Fig. 1a, b) and the soma. By construction, for the passive case, the transfer resistance (for *ω* = 0) is equivalent for the respective loci in the detailed and the reduced model. This is indeed the case in Fig. 1d (left-most point on the *x*-axis), thus validating the implementation of the Neuron_Reduce analytic method. Note that although constructed using *ω* = 0, the similarity between the detailed and reduced model also holds for higher input frequencies. However, for *ω* around 10–100 Hz, the transfer impedance from *d* to soma (and vice versa, due to the reciprocity theorem for passive systems^43^) is somewhat larger in the reduced model (compare the red and black lines).

To test the performance of Neuron_Reduce on transient synaptic inputs (composed of mixed input frequencies), we sequentially activated the four synapses shown in Fig. 1c in both the detailed and the reduced models (see Methods and Supplementary Table 2). Figure 1e shows the close similarity in the composite somatic EPSPs between the two models, further validating that the mapping of the detailed model to the reduced model using *ω* = 0 provides satisfactory results for the passive case (see also Supplementary Fig. 2).

### Accuracy and speed-up of Neuron_Reduce for nonlinear models

**Fig. 2:**
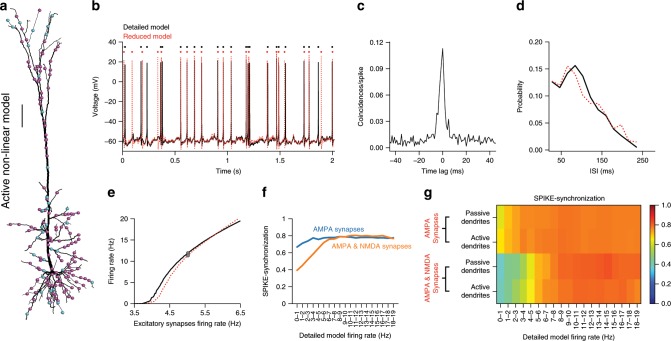
Neuron_Reduce faithfully replicated the I/O properties of a detailed nonlinear model of a L5 pyramidal cell. **a** Layer 5 pyramidal cell model^5^ as in Fig. 1a, with 8000 (AMPA + NMDA) excitatory (magenta dots) and 2000 inhibitory synapses (cyan dots, see Supplementary Table 2 for synaptic parameters). Excitatory synapses were activated randomly at 5 Hz and the inhibitory synapses at 10 Hz. This detailed model consists of a dendritic Ca^2+^ “hot spot” (as in Fig. 1c) and a Na^+^ spiking mechanism at the cell body. Scale bar 100 µm. **b** An example of the voltage dynamics at the soma of the detailed model (black trace) and the reduced model (red trace); spike times are represented by the black and red dots above the respective spikes. **c** Cross-correlation between spikes in the reduced versus the detailed models. **d** Inter-spike interval (ISI) distributions for the two models. **e** Output firing rate of the reduced (red) versus the detailed (black) models as a function of the firing rate of the excitatory synapses. Gray dots represent the case shown in **b**. **f** SPIKE-synchronization measure between the two models as a function of the firing rate of the detailed model for the case of only AMPA (blue) and AMPA + NMDA synapses (orange). The performance of the reduced model with NMDA synapses was lower for low output frequency, but improved significantly for output frequencies above ~7 Hz (see Discussion). **g** SPIKE synchronization between the detailed and the reduced models as a function of the firing rate of the detailed model, for active and passive dendrites, and with/without NMDA-based synaptic conductance.

To measure the accuracy of Neuron_Reduce for a fully-active nonlinear neuron model, we ran a comprehensive set of simulations using the well-established case of the L5 pyramidal cell model^5^ shown in Fig. 2a (same cell as in Fig. 1). This neuron model includes a variety of nonlinear dendritic channels including a voltage-dependent Ca^2+^ “hot spot” in the apical tuft (schematic yellow region in Fig. 1c) and a Na^+^-based spiking mechanism in the cell body. We randomly distributed 8000 excitatory and 2000 inhibitory synapses on the modeled dendritic tree (the synaptic parameters are listed in Supplementary Table 2) and used Neuron_Reduce to generate a reduced model for this cell. We simulated the detailed model by randomly activating the excitatory synapses at 5 Hz and the inhibitory synapses at 10 Hz (see Methods). The detailed model responded with an average firing rate of 11.8 Hz (black trace in Fig. 2b; only 2 out of 50 s simulation time are shown). The average firing rate of the respective reduced model in response to the same synaptic input was 11.3 Hz (red trace, Fig. 2b; spike timings are shown by small dots on the top). The cross-correlation between the two spike trains peaked around zero (Fig. 2c), and the inter-spike interval (ISI) distributions of the two models were similar (Fig. 2d).

The full range of responses to a random synaptic input for the two models was explored by varying the firing rate of the excitatory (α-amino-3-hydroxy-5-methyl-4-isoxazolepropionic acid (AMPA)- and (*N*-methyl-d-aspartate) (NMDA)-based) synapses and measuring the degree of similarity between the firing rates of the two models, which indicated a good fit between the two (Fig. 2e). We used the SPIKE-synchronization measure^44,45^ to further quantify the similarity between the spike trains of the detailed and reduced models. The SPIKE-synchronization value for the two spike trains shown in Fig. 2b was 0.8. In Fig. 2f, the SPIKE synchronization was computed as a function of the output rate of the detailed model for both the case where the excitatory synapses consisted of only an AMPA component (blue) and for when they also consisted of an NMDA component (orange). For the AMPA-only case, the SPIKE synchronization was high for all output frequencies, but was poor for low output frequencies when the synapses consisted of an NMDA component, although improving significantly for output frequencies above ~7 Hz (see Discussion). Figure 2g shows the SPIKE-synchronization as a function of the firing rate of the detailed model, for active and passive dendrites and with/without NMDA-based synaptic conductance, demonstrating again that when NMDA synapses are involved, the performance of the reduced model is low for low output rates. We also tested other spike trains similarity metrics^46,47^ (Supplementary Fig. 3) and found comparable results to those shown in Fig. 2. We have also analyzed the performance of Neuron_Reduce on two additional patterns of synaptic input. In one case, the synaptic input was activated in an oscillatory manner at different frequencies (see Methods). In these cases, the spike-synchronization measure ranged between 0.75 and 1 (Supplementary Fig. 4a, b). In the other case, the synaptic input was taken from a spontaneously active Blue Brain circuit^17^ (see Methods). In this case, the spike-synchronization measure was 0.71 (Supplementary Fig. 4c).

We compared the performance of our reduction method to two other reduction approaches, one of which was Rall’s “equivalent cable” reduction method^31,48^. The other method maps all the dendritic synapses to the somatic compartment, after computing the filtering effect of the dendritic cable for each synapse^34^ (see Methods). Neuron_Reduce outperformed both these reduction methods (Supplementary Fig. 5).

**Fig. 3:**
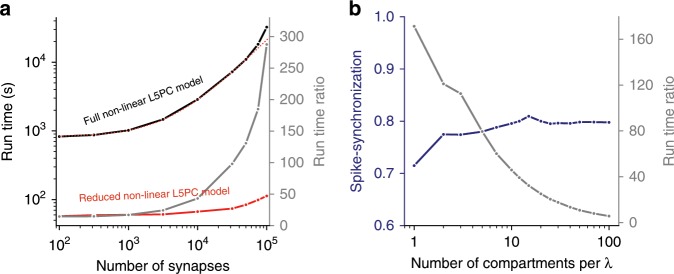
Neuron_Reduce enhances the simulation speed by up to several hundred fold. **a** Simulation run-time for the detailed (black) and the reduced models (red) of layer 5 pyramidal cell shown in Fig. 2a, for a simulation of 50 s, and their ratio (the speed-up, gray) as a function of the number of simulated (GABAA-, AMPA- and NMDA- based) synapses. Due to the almost constant run-time of the reduced model, the run-time ratio increases with larger number of synapses. Above 75,000 synapses, an additional effect becomes visible: the detailed model no longer fits into the cache of the CPU and exhibits a supralinear increase in run-time. This can be seen by the black curve deviating from the dotted red curve, which shows the expected simulation time for the detailed model assuming a constant computation cost per synapse (see also Supplementary Table 1). **b** Accuracy (blue) of the reduced model and its speed-up in simulation run-time (gray) as a function of the number of electrical compartments per length constant for a neuron with 10,000 synapses (50 s per simulation).

**Fig. 4:**
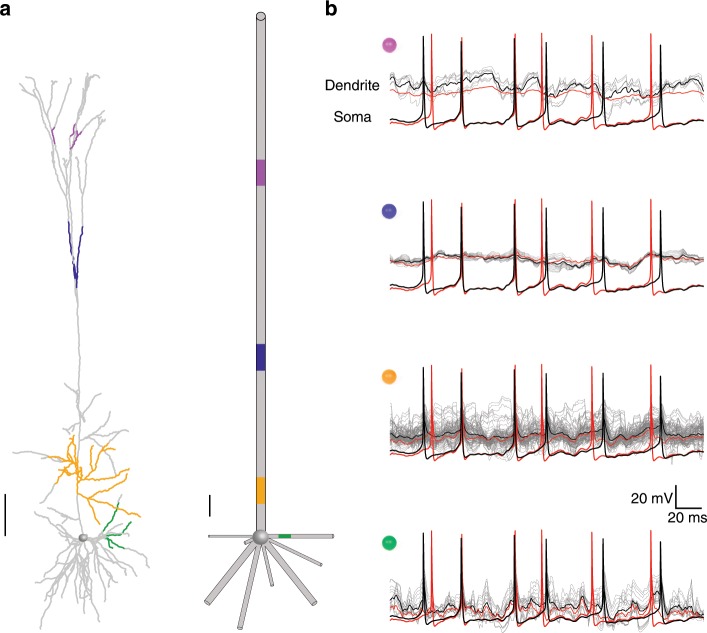
The dendritic potential in the reduced model represents the average dendritic voltage dynamics in the detailed model. **a** Detailed model (left) and reduced model (right) of the cell shown in Fig. 2. Dendritic branches of the same color in the detailed model are all mapped to the respective compartment with identical color in the reduced model. **b** For each of the four colored regions shown in **a** (and respective colored sphere at top left), the voltage transients in individual branches are shown by the gray traces. Superimposed in black is the average voltage of these traces and in red is the voltage transient in the respective compartment in the reduced model. The somatic spikes in the detailed model (black) and reduced model (red) are also shown. The simulation is as in Fig. 2e, with excitatory synapses firing at 5.5 Hz. Scale bars for the respective morphologies are 100 µm.

**Fig. 5:**
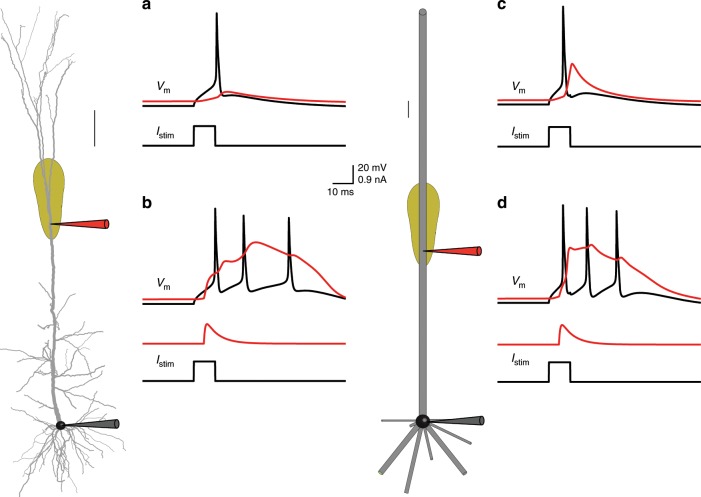
Dendritic Ca^2+^ spike and BAC firing faithfully replicated in the reduced model. **a**, **b** (Left) Detailed L5 pyramidal cell model with nonlinear Ca^2+^ “hot spot” (same model as in Fig. 2). **a** Injecting a depolarizing step current to the soma (0.95 nA for 8.5 ms) in the detailed model evoked a somatic action potential, AP (black trace) that propagated backward semi-actively into the apical tree (red trace). **b** Combining the somatic input with a transient synaptic-like current injection (0.95 nA peak value with 0.5 and 5 ms rise time and decay time, respectively; red transient) to the “hot region” in the apical dendrite evoked a prolonged local Ca^2+^ spike, which, in turn, triggered a burst of two extra somatic Na^+^ spikes (the BAC firing phenomenon^50^). **c**, **d** Same as in **a**, **b**, but for the reduced model. Scale bars for the respective morphologies are 100 µm.

**Fig. 6:**
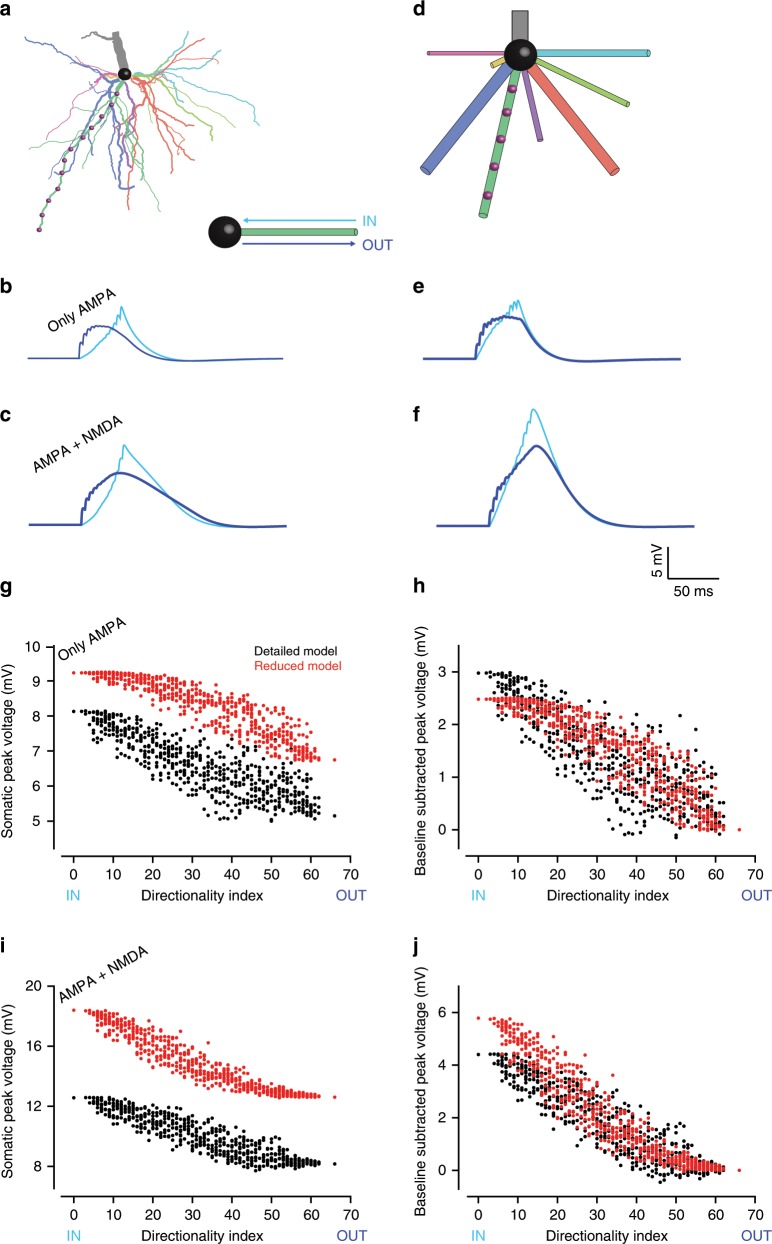
Discriminating spatio-temporal input sequences in the detailed versus the reduced model. **a** A model of L5PC (detailed model, Fig. 1) with 12 excitatory synapses spatially distributed on one of its basal dendrites (red dots on green basal dendrite). **b** Somatic responses to sequential activations of its basal synapses in the IN (cyan) and the OUT (blue) directions. In this case, the synaptic model only consists of an AMPA component. **c** As in **b** but the synaptic model consists of both AMPA and NMDA components. **d** Reduced model for the detailed model shown in a. Neuron_Reduce mapped the 12 synapses in the detailed model into five synapses in the reduced model. **e**, **f**. As in **b**, **c**, but for the reduced model. **g** Pattern separability (see Methods) of the detailed (black) and the reduced (red) models when the synaptic model only consists of an AMPA component. **h** As in **g**, after subtracting the peak voltage obtained in the OUT direction from each of the voltage responses. **i**, **j** As in **g**, **h** but when the synaptic models consisted of both AMPA and NMDA conductances. Note the similarity between the detailed and the reduced models in terms of pattern separability.

**Fig. 7:**
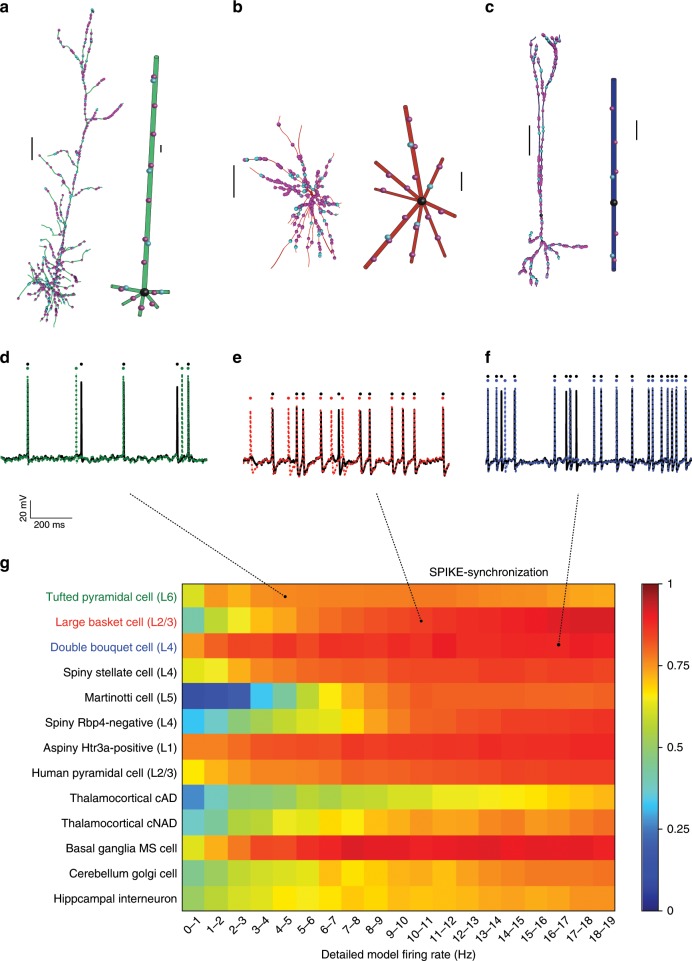
Neuron_Reduce working successfully on a variety of neuron models. **a**–**c** Detailed models of three somatosensory neurons (left, L6 tufted pyramidal cell in green; middle, L2/3 large basket cell in red; and right, L4 double bouquet cell in blue) and their respective reduced models. Scale bars 100 µm. **d**–**f** Voltage responses to an excitatory synaptic input activated at 1.8, 2.9, and 3.17 Hz, respectively, for both the detailed (black) and the reduced models (corresponding colors). The inhibitory input activation rate was 10 Hz for all models. **g** The SPIKE-synchronization index for the 13 detailed versus reduced neuron models. The mean simulation speed-up for the L6 tufted pyramidal cell, L5 Martinotti cell, and L4 spiny stellate cell were 95, 40, and 60, respectively. See Supplementary Table 2 for cell models and input parameters and Supplementary Fig. 6 for the SPIKE-synchronization measure on additional 88 modeled cells.

Figure 3 compares the run-time of the detailed versus the reduced model for the neuron model shown in Fig. 2a. For example, simulating the detailed model with 10,000 synapses for 50 s of biological time required 2906 s of computer time (run-time), whereas it took only 68.7 s in the reduced model, a ~42-fold computational speed-up (see Supplementary Table 1). The larger the number of synapses in the detailed model, the longer the run-time (Fig. 3a). In contrast, the run-time in the reduced model is only shallowly dependent on the number of synapses. This is expected when considering the synaptic merging step in our algorithm (see Discussion). The run-time of the reduced model depends on the number of compartments per cylinder; it increases sharply with an increasing number of compartments (the run-time ratio between the detailed and the reduced models decreases, gray line in Fig. 3b). However, there was no improvement in the SPIKE-synchronization measure when the spatial discretization, Δ*X*, per compartment was <0.1*λ*, where λ is the length constant (Fig. 3b blue line and see also previous research on the subject^49^). Therefore, all the results presented in Figs. 1–7 are based on models with a Δ*X* that does not exceed 0.1*λ*.

In Fig. 4 we compared the dendritic voltage in the detailed model and in the respective location in the reduced model. We found that: (i) the voltage transients could differ significantly in dendritic branches that are all mapped to the same compartment in the reduced model (e.g., compare the gray traces in the yellow compartments in Fig. 4b). (ii) the average voltage trace of these different dendritic branches (black trace in Fig. 4b) is similar to the voltage in the respective compartment in the reduced model (red trace in Fig. 4b). The implications of the latter finding for capturing highly nonlinear local dendritic events is elaborated in the Discussion.

### Neuron_Reduce keeps dendritic nonlinearities and computations

To determine the capabilities of the reduced models to support nonlinear dendritic phenomena and dendritic computations, we repeated two classical experiments in both the detailed and the reduced models of the L5 pyramidal cell shown in Fig. 1. The first simulated experiment started by injecting a brief depolarizing step current to the soma of the detailed model to generate a somatic Na^+^ action potential (AP, black trace in Fig. 5a). This AP propagated backward to the apical dendrite, the BPAP (red trace in Fig. 5a). Repeating the same current injection in the reduced model led to a similar phenomenon, but with a larger BPAP (Fig. 5c). The detailed model also included a “hot region” with voltage-dependent calcium conductances in its apical dendrite (see also Fig. 1). Combining somatic current injection with synaptic-like transient depolarizing current injected to the apical nexus evoked a prolonged Ca^2+^ spike in the distal apical dendrite (red trace at the apical tree), which, in turn, generated a burst of somatic Na^+^ spikes (the BPAP-activated Ca^2+^ spike (BAC) firing^4,5,50^, Fig. 5b). Neuron_Reduce maps the nonlinear dendritic “hot” Ca^2+^ region to its respective location in the reduced model (see Fig. 1 and Methods). Figure 5c, d shows that the exact same combination of somatic and dendritic input currents also produced the BAC firing phenomenon in the reduced model. However, the reduced model was somewhat more excitable than the detailed model; this resulted in a burst of three spikes with a higher frequency (and sometimes with an additional spike) in the reduced model (comparison between Fig. 5b–d).

The second simulated experiment attempted to replicate theoretical and experimental results reported in previous studies^1,51,52^. In these studies, several excitatory synapses were activated sequentially in time, on a stretch of a basal dendrite, either in the soma-to-dendrites (OUT) direction or vice versa (the IN direction). Rall showed that the shape and size of the resultant composite somatic EPSP depended strongly on the spatio-temporal order of synaptic activation; it was always larger and more delayed for the centripetal (dendrites-to-soma) than for the centrifugal (soma-to-dendrites) sequence of synaptic activation (this difference can serve to compute the direction of motion^51^). It was shown that the difference in the resulting somatic voltage peak between these two spatio-temporal sequences of synaptic activation was enhanced when nonlinear NMDA-dependent synapses were involved and that it made it possible to discriminate between complex patterns of dendritic activation^52^.

To simulate these phenomena, 12 excitatory synapses were placed along one basal branch in the detailed model (red dots on the green basal tree, Fig. 6a). At first, the synapses only had an AMPA component. The synapses were activated in temporal order from the tip to the soma (IN, cyan traces) or from the soma to the tip (OUT, blue traces, see Methods for details). As predicted by Rall, activation in the IN direction resulted in larger and delayed somatic EPSP (cyan trace versus the blue trace in Fig. 6b). Neuron_Reduce merged these 12 synapses into five point processes along the respective cylinder (Fig. 6d). We repeated the same experiment in the reduced model and found that the EPSP resulting from the IN direction was larger and delayed, with a similar EPSP waveform to that of the detailed model (Fig. 6e; see also Supplementary Fig. 2 and Discussion). Next, an NMDA component was added to the 12 simulated synapses; this resulted in larger somatic EPSP amplitudes in both directions (and both models) and a smaller difference in the peak timing between the different directions in both the detailed and the reduced models (comparison between Fig. 6c–f).

To generalize the impact of the spatio-temporal order of synaptic activation, we used a directionality index suggested in a previous study^52^. This measure estimates how different a given synaptic sequence is from the IN sequence by calculating the number of synaptic swaps needed to convert this given pattern into the IN pattern (using the bubble-sort algorithm, see Methods). We tested the EPSPs that resulted from different temporal combinations of synaptic activation (each having a different directionality index), both without (Fig. 6g) and with an NMDA component (Fig. 6i). The peak somatic EPSP in the reduced model (red dots) was larger than in the respective detailed model (black dots), both for the AMPA-only case (by 1.71 ± 0.43 mV; mean ± SD) and for the AMPA + NMDA case (by 4.80 ± 0.74 mV); see Supplementary Fig. 1. Nevertheless, the behavior of the two models was similar when the somatic voltage in the two models was subtracted by the peak value obtained in the OUT direction (Fig. 6h, j). Then, the difference between the reduced and the detailed models was, on average, only 0.11 ± 0.43 mV for the AMPA-only case and 0.35 ± 0.74 mV for the AMPA + NMDA case. Thus, although the detailed and the reduced models differ to a certain extent (see Discussion), the capability of the reduced model to discriminate between spatio-temporal patterns of synaptic activation is similar to that of the detailed model.

### Neuron-Reduce applied successfully on a variety of neurons

We next tested the utility of Neuron_Reduce on 13 different neuron models from different brain regions (Fig. 7). Four models were obtained from the Blue Brain database^17,53^: L6 tufted pyramidal cell, L4 double bouquet cell, L4 spiny stellate cell, and L5 Martinotti cell, all from the rat somatosensory cortex. Two additional models were obtained from the Allen Institute cell-type database^11^: an L4 spiny cell and an L1 aspiny cell from the mouse visual cortex. Medium spiny neuron from the mouse basal ganglia^54^; two rat thalamocortical neurons^55^; Golgi cell from mouse cerebellar cortex; and one inhibitory hippocampal neuron from the rat^56^. We also took two additional neuron models from our laboratory: rat L2/3 large basket cell^57^ and a model of a human L2/3 pyramidal cell from the temporal cortex^58^. All these models were based on 3D reconstructions and were constrained by experimental recordings (see Supplementary Table 2 for details on the various neuron models and input parameters).

Neuron_Reduce successfully generated a reduced model for all these different cell types, with highly faithful response properties in all cases (Fig. 7). Three examples with their respective morphologies for the detailed and reduced models are shown in Fig. 7a–c. For a given input, we measured the spiking activity of the detailed and reduced models (Fig. 7d–f) and calculated the corresponding SPIKE-synchronization values. For the L6 tufted PC model (Fig. 7a, d), the L2/3 large basket cell model (Fig. 7b, e), and the L4 double bouquet model (Fig. 7c, f), the SPIKE-synchronization values were 0.74, 0.85, and 0.91, respectively, for 50-s-long simulations (only 2 s are shown in Fig. 7d–f). The SPIKE-synchronization values for additional inputs, and for the other 10 neuron models and their corresponding reduced models, are shown in Fig. 7g. We have also tested the performance of Neuron_Reduce and the variability of the SPIKE-synchronization measure using eight neocortical neuron types, with 11 cell models per type taken from the Blue Brain cells dataset^17,53^. Supplementary Fig. 6 shows that, for all cells, the SPIKE-synchronization measure remains similar to that found in Fig. 7 with mean values per cell type ranging between 0.43 and 0.86. Additionally, as in Fig. 7, it increased  with the output frequency of the modeled cell.

## Discussion

Neuron_Reduce is a new tool for simplifying complex neuron models while enhancing their simulation run-time. It analytically maps the detailed tree into a reduced multi-cylindrical tree, based on Rall’s cable theory and linear circuit theory (Fig. 1). The underpinning of the reduction algorithm is that it preserves the magnitude of the transfer impedance $| {Z_{0,j}\left( \omega \right)} |$ from each dendritic location, *j*, to the soma (the dendro-somatic direction, Eqs. (1)–(11) in Methods). Since in linear systems it holds that $| {Z_{0,j}(\omega )} | = | {Z_{j,0}(\omega )} |$, for passive dendritic trees it also preserves the transfer impedance in the soma-to-dendritic direction (e.g., current injection at the soma will result in the same voltage response at the respective sites in the detailed and reduced models^59^).

Note that dendritic voltage transients (e.g., synaptic potentials) contain a range of frequencies, *ω*. We however had to select one frequency to use for the mapping of the detailed-to-the-reduced tree. Consequently, we examined a whole range of possible *ω* values for this mapping. Conveniently, we found that *ω* = 0 is the preferred frequency for generating the reduced model (namely, when the mapping from detailed-to-the-reduced model is performed based on the transfer resistance $| {Z_{0,j}\left( {\omega = 0} \right)} | = | {R_{0,j}} |$, see Supplementary Fig. 7). This result is actually not surprising; Rinzel and Rall^33^ showed that, in passive trees and current-based synapses, the attenuation of the voltage time integral (the area below the EPSPs) is identical to the attenuation of steady-state voltage. In other words, when using the transfer resistance for our mapping procedure, we preserved the total charge transfer (which in our case, was proportional to the voltage time integral) from the synapse to the soma (and vice versa), but not, for example, the EPSP peak value.

Neuron_Reduce was proven to be accurate in replicating voltage dynamics and spike timing for a large regime of input parameters and a variety of neuron types (Fig. 7, Supplementary Fig. 6, and Supplementary Table 2). This claim is based on using several metrics for assessing the quality of the performance of the reduced model (Supplementary Fig. 3). Neuron_Reduce is straightforward to use, it is fast, and generally applicable, thus enabling its implementation on any neuron morphology with any number (even tens of thousands) of synapses. One key advantage of Neuron_Reduce is that it retains the identity of individual dendrites and synapses and that it maps dendritic nonlinearities to their respective loci in the reduced model, hence preserving local excitable dendritic phenomena and therefore maintaining nonlinear dendritic computations. Neuron_Reduce also preserves the passive cable properties (*R*_m_, *R*_a_, and *C*_m_) of the detailed model, thus preserving synaptic integration and other temporal aspects of the detailed model. Neuron_Reduce can also be applied for reducing cells connected with gap junctions. As Neuron_Reduce preserves the transfer resistance from the location of the synapses (in this case the gap junction) to the soma and vice versa, one expects that the coupling coefficient between the two connected cells will be preserved in the reduced models, after mapping the gap junction to its appropriate location in the reduced model.

Neuron_Reduce enhances the computational speed by a factor of up to several hundred folds, depending on the simulated morphology and the number of simulated synapses (Fig. 3 and Supplementary Table 1). This combination of capabilities, together with its user-friendly documentation and its public availability, make Neuron_Reduce a promising method for the community of neuronal modelers and computational neuroscientists, and for the growing community interested in “biophysical deep learning.”

For a large number of synapses and complex morphologies, the run-time of Neuron_Reduce models can be accelerated by up to 250-fold as compared to their respective detailed models (Fig. 3 and Supplementary Table 1). This is achieved in two associated steps. First, the algorithm reduces the number of compartments of the neuron model; for example, for the reconstructed tree in Fig. 1, it reduced the number of compartments from 642 to 50. Then, synapses (and ion channels) that are mapped to the same electrical compartment in the reduced tree (because they have similar transfer resistance to the soma) are merged into one point process in NEURON. Each of these steps on its own has a relatively small effect on the run-time. However, when combined, a large (supralinear) improvement in the computational speed is achieved (Supplementary Table 1). This is because at each time step, NEURON computes both the voltage in each electrical compartment as well as the currents and states of each point process and membrane mechanism (synapses and conductances). Reducing the number of compartments in a model decreases the number of equations to be solved and the number of synapses to be simulated (due to the reduced number of compartments, a larger number of synapses are merged together). Importantly, merging synapses preserves the activation time of each synapse. Note, however, that in its present state, Neuron_Reduce cannot merge synapses with different kinetics.

Several other reduction methods for single neurons have been proposed over the years^12,34–39,41^. Most are not based on an analytic underpinning and thus require hand-tuning of the respective biophysical and morphological parameters. In addition, most of these methods have not been examined using realistic numbers of dendritic synapses and are incapable of systematic incorporation of dendritic nonlinearities. In most cases, their accuracy has not been assessed for a range of neuron types (but see ref. ^41^). Many of these methods are not well documented, thus making it hard to compare them directly with Neuron_Reduce. Nevertheless, we did compare the performance of Neuron_Reduce to two other reduction methods and showed that it outperformed them (Supplementary Fig. 5).

It should be noted that although the transfer impedance from a given dendritic locus to the soma is preserved in the reduced model, the input impedance at that locus is not preserved (is lower) in the reduced model. Consequently, the conditions for evoking local dendritic events, and the fine details of these events are not identical in the detailed and the reduced models (e.g., compare Fig. 5a, b to Fig. 5c, d and see Fig. 4). Indeed, if there were highly local dendritic Na^+^ spikes (as in ref. ^60^), then Neuron_Reduce will not capture them, as this local dendritic spike will be averaged out in the respective lumped cable. Similarly, because the local voltage response to a current injection in the dendrite depends on the dendritic impedance, the local synaptic responses are somewhat different in the detailed versus the reduced cases, especially when voltage-gated ion channels (such as NMDA-dependent synaptic channels) are involved. In fact, when large dendritic NMDA signals are involved, the resultant somatic EPSPs are expected to be different in the detailed as compared to the reduced model, as is the case in Figs. 2 and 6. Indeed, if one insists on preserving highly local nonlinear dendritic events, then the full dendritic tree should be modeled.

Despite these local differences, the reduced model for L5PC did generate a local dendritic Ca^2+^ spike in the cylinder representing the apical dendrite and was able to perform an input classification task (enhanced by NMDA conductance), as in the detailed tree (Figs. 5 and 6). Moreover, when embedded in large circuits, individual neurons are likely to receive semi-random dendritic input, rather than a clustered input on specific dendrites. For such inputs, the reduced models generated by Neuron_Reduce capture most of the statistics of the membrane voltage dynamics as in the detailed model (Figs. 2 and 7 and Supplementary Figs. 4 and 6).

The next straightforward step is to use Neuron_Reduce to simplify all the neurons composing a large neural network model, such as the Blue Brain Project^17^ and the in silico models by Egger et al.^16^ and by Billeh et al.^61^. By preserving the connectivity and reducing the complexity of the neuronal models, the reduced models will make it possible to run much longer simulations and/or larger neuronal networks, while faithfully preserving the I/O of each neuron. Such long simulations are critical for reproducing long-term processes such as circuit evolution and structural and functional plasticity.

## Methods

### Neuron_Reduce algorithm and its implementation in NEURON

Neuron_Reduce maps each original stem dendrite to a unique single cylinder with both ends sealed. This cylinder preserves the specific passive cable properties (*R*_m_, *C*_m_, and *R*_a_) of the original tree as well as both the transfer impedance from the electrotonically most distal dendritic tip to the soma and the input resistance at the soma end of the corresponding stem dendrite (when disconnected from the soma). For a sinusoidal angular frequency *ω* > 0, the transfer impedance *Z*_*i,j*_(*ω*) is the ratio between the Fourier transform of the voltage at point (*i*) and the Fourier transform of the sinusoidal current injected into the injection point (*j*) (note that in passive systems, *Z*_*i,j*_(*ω*) = *Z*_*j*,*i*_(*ω*)). This ratio is a complex number; its magnitude (|*Z*_*i*,*j*_(*ω*)|) is the ratio (in Ω) between the peak voltage response and the amplitude of the injected current. In a short cylindrical cable with sealed ends and electrotonic length *L*, the transfer impedance, *Z*_0*,X*_(*ω*), between the somatic end of the cylinder (*X* = 0) and any location *X* is^33,43,62^

1 $$Z_{0,X}\left( \omega \right) = \frac{{R_\infty }}{q}\frac{{{\mathrm{cosh}}\left( {q\left( {L - X} \right)} \right)}}{{{\mathrm{sinh}}(qL)}},$$

where

2 $$R_\infty = \frac{2}{\pi }\frac{{\sqrt {R_{\mathrm{m}}R_{\mathrm{a}}} }}{{d^{3/2}}}$$

and

3 $$q = \sqrt {1 + i\omega \tau },$$

where *τ* is the membrane time constant, *R*_m_*C*_m_.

From Eq. (1), the input impedance at *X* = 0 is

4 $$Z_{0,0}\left( \omega \right) = \frac{{R_\infty }}{q}{\mathrm{coth}}(qL).$$

We next want a cylindrical cable of electrotonic length *L*, in which both $| {Z_{0,L}\left( \omega \right)} |$ and $| {Z_{0,0}\left( \omega \right)} |$ are identical to those measured in the respective original stem dendrite (Fig. 1). For this purpose, we first look for an *L* value in which the ratio $| {Z_{0,L}\left( \omega \right)} |/| {Z_{0,0}\left( \omega \right)} |$ is preserved. Dividing Eq. (1) by Eq. (4), we get

5 $$\frac{{Z_{0,X}\left( \omega \right)}}{{Z_{0,0}\left( \omega \right)}} = \frac{{\cosh \left( {q(L - X)} \right)}}{{\cosh \left( {qL} \right)}},$$

which can be expressed as

6 $$\frac{{Z_{0,X}\left( \omega \right)}}{{Z_{0,0}\left( \omega \right)}} = \frac{{{\mathrm{cosh}}\left( {a\left( {L - X} \right) + ib\left( {L - X} \right)} \right)}}{{{\mathrm{cosh}}(aL + ibL)}} = {M{\mathrm{exp}}}(i\phi ),$$

where *a* and *b* are the real and the imaginary parts of *q*, respectively, and *M* and *ϕ* are the modulus and phase angle of this complex ratio.

As shown previously^62^, it follows that

7 $${M} = \frac{{| {Z_{0,X}\left( \omega \right)} |}}{{| {Z_{0,0}\left( \omega \right)} |}} = \left[ {\frac{{\cosh \left( {2a\left( {L - X} \right)} \right) + \cos \left( {2b\left( {L - X} \right)} \right)}}{{\cosh \left( {2aL} \right) + \cos \left( {2bL} \right)}}} \right]^{0.5}$$

and

8 $$\phi = \arctan \left[ {\tanh \left( {a\left( {L - X} \right)} \right)\tan \left( {b\left( {L - X} \right)} \right)} \right] - \arctan \left[ {\tanh \left( {aL} \right)\tan \left( {bL} \right)} \right].$$

Importantly, for a fixed *M* (and a given *ω*) there is a unique value of *L* that satisfies Eq. (7) (see Fig. 4 in ref. ^62^ and note the-one-to-one mapping between *M* and *L* for a given *ω* value). However, there are an infinite number of cylindrical cables (with different diameters and lengths) that have identical *L* values preserving a given *M* value in Eq. (7).

We next need a unique cable, with a specific diameter *d*, that also preserves the measured value of |*Z*_0,0_(*ω*)| (and therefore it also preserves |*Z*_0,*L*_(*ω*)|, see Eq. (7)).

From Eqs. (2) and (4) we get

9 $$Z_{0,0}\left( \omega \right) = \frac{2}{{\pi q}}\frac{{\sqrt {R_{\mathrm{m}}R_{\mathrm{a}}} }}{{d^{3/2}}}{\mathrm{coth}}(qL)$$

and thus

10 $$| {Z_{0,0}\left( \omega \right)} | = \left| {\frac{2}{{\pi q}}\frac{{\sqrt {R_{\mathrm{m}}R_{\mathrm{a}}} }}{{d^{3/2}}}{\mathrm{coth}}(qL)} \right|$$

from which we compute the diameter, *d*, for that cylinder

11 $$| d | = \left| {\left( {\frac{2}{\pi }\frac{{\sqrt {R_{\mathrm{m}}R_{\mathrm{a}}} }}{{qZ_{0,0}\left( \omega \right)}}\coth \left( {qL} \right)} \right)^{2/3}} \right|.$$

Equations (1)–(11) provide the unique cylindrical cable (with a specific *d* and *L*, and the given membrane and axial properties) that preserves the values of $| {Z_{0,L}\left( \omega \right)} |$ and $| {Z_{0,0}\left( \omega \right)} |$ as in the respective stem dendrite. Note that this unique cable does not necessarily preserve the phase ratio (*ϕ* in Eq. (8)) as in the original tree.

Practically, in order to transform each original stem dendrite (with fixed *R*_m_, *R*_a_, and *C*_m_ values) into a corresponding unique cylindrical cable, we proceeded as follows. First, on each modeled stem dendrite (when isolated from the soma), we searched for a distal location *x* with minimal transfer impedance, $| {Z_{0,x}\left( \omega \right)} |$, from that particular *x* to the soma. This location provided the smallest *M* value for this particular stem dendrite. This distal dendritic locus, *x*, was mapped to the distal end, *X* = *L*, of the corresponding cylinder. We then used Eqs. (1)–(11) to calculate the unique respective cylinder for each stem dendrite.

In order to map synapses from the detailed model to the reduced one, we computed, for each synapse at location *j* in the detailed model, $| {Z_{0,j}\left( \omega \right)} |$, and then mapped this synapse to the respective location *y* in the reduced model, such that $| {Z_{0,y}\left( \omega \right)} | = | {Z_{0,j}\left( \omega \right)} |$. This reduced model is then compartmentalized into segments (typically with spatial resolution of 0.1*λ*, see Fig. 3b). We then merged all synapses with identical kinetics and reversal potential, that are mapped to a particular segment, onto a single-point process object in NEURON (Fig. 1, Step B). These synapses retain their original activation time and biophysical properties through the connection of each of their respective original NetStim objects to the single-point process that represents them all (each of these connections was mediated by the synapse’s original NetCon object). As shown in Supplementary Table 1, this step dramatically reduced the running time of the model. We note that all the results presented in this study were obtained using *ω* = 0 in Eqs. (1)–(11), since running the same simulations with *ω* = 0 provided the best performance (see Supplementary Fig. 7). However, *ω* is a parameter in the algorithm code and can be modified by the user. Note also that $| {Z_{0,0}\left( \omega \right)} |$, $| {Z_{0,j}\left( \omega \right)} |$, and $| {Z_{0,L}\left( \omega \right)} |$ were analytically computed for each original stem dendrite using the NEURON impedance tool^63^.

### Neuron models used in the present study

To estimate the accuracy of the reduction method, we ran 50-s simulations of cell morphologies of different types, in both the reduced and detailed models (see also Supplementary Fig. 6). Models of 13 neurons were used in this study; their details are available in Supplementary Table 2. For each of the models, we distributed 1,250–10,000 synapses on their dendritic trees. Eighty percent of the synapses were excitatory, and the rest were inhibitory. The synaptic conductances were modeled using known two-state kinetic synaptic models^17^. For simplicity, we did not include synaptic facilitation or depression. All models had one type of γ-aminobutyric acid type A (GABA_A_)-based inhibitory synapses and either AMPA- or AMPA + NMDA-based excitatory synapses. The synaptic rise and decay time constants were taken from various works cited in Supplementary Table 2. When no data were available, we used the default parameters of the Blue Brain Project synaptic models^17,53^. Inhibitory synapses were activated at 10 Hz, whereas the activation rate of the excitatory synapses was varied to generate different output firing rates in the range of 1–20 Hz (Figs. 2–4, 7 and Supplementary Figs. 3–7); the values used for each model are listed in Supplementary Table 2. In all models except Supplementary Fig. 4, synaptic activation time was randomly sampled from a homogenous Poisson process. In Supplementary Fig. 4a, b the activation time was sampled from an inhomogeneous Poisson process with a time-dependent intensity $\lambda \left( t \right) = r \, \ast \, {\mathrm{sin}}\left( {t \, \ast f \ast 2\pi } \right) + 1$, where *t* is time in s, *r* is the firing rate of the synapse, and *f* is the oscillation frequency.

In Supplementary Fig. 4c, we extracted a single-layer five thick-tufted pyramidal cell with an early bifurcating apical tuft (L5_TTC2; gid 75586) from active blue brain microcircuit^17^ with calcium and potassium concentration of 1.23 and 5.0 mM, respectively. The synaptic activation from the microcircuit was replayed to this detailed model and also to its respective reduced model. Synaptic depression and facilitation were disabled, and the synapse time constants, which varied in the microcircuit, were set to their mean value (the decay time constant was set to 1.74 and 8.68 ms for AMPA and GABA_A_, respectively; the rise time constant for GABA_A_ was set to 4.58 ms); all other variables were as in the blue brain simulations.

### Estimating the accuracy of the reduced models

Cross-correlations were calculated between the spike trains of the detailed and the reduced models. The window size was 500 ms, and the bin size was 1 ms. The resulting cross-correlations were normalized by the number of spikes in the detailed model (Fig. 2c). ISIs were binned in windows of 21 ms to create the ISI distribution in Fig. 2d.

SPIKE-synchronization measure is a parameter- and scale-free method that quantifies the degree of synchrony between two spike trains^44^. SPIKE-synchronization uses the relative number of quasi-simultaneous appearances of spikes in the spike trains. In this study, we used the Python implementation of this method^64^. To allow comparison to the literature, Supplementary Fig. 3 depicts three additional metrics against which to compare the performance of the detailed and the reduced models: Trace accuracy^39^, ISI distance^44^, and *Γ* coincidence factor^65^.

### Comparison to other reduction algorithms

We compared Neuron_Reduce to two classical reduction algorithms (Supplementary Fig. 5):

1. Equivalent cable using the d3/2 rule for reduction. Rall and Rinzel^32^ and Rinzel and Rall^33^ showed that for idealized passive dendritic trees, the entire dendritic tree can be collapsed to a single equivalent cylinder that is analytically identical (from the point of view of the soma) to the detailed tree. However, neurons do not have ideal dendritic trees, mostly because dendritic terminations typically occur at different electrotonic distances from the soma. Nevertheless, it is possible to collapse any dendritic tree using a similar mapping (Rall’s “d3/2 rule”) as in the idealized tree; this will provide an “equivalent cable” (rather than an “equivalent cylinder”) with a varying diameter for the whole dendritic tree (see details in Rall et al.^48^). The electrotonic distances to the soma of synapses and nonlinear dendritic mechanisms were computed in the original model and then each synapse and mechanism was mapped to the corresponding segment in the “equivalent cable” preserving the electrotonic distance to the soma as in the original tree.
2. Mapping all synapses to the soma. Another recent reduction scheme was proposed where all dendritic synapses are mapped, after implementing cable filtering for each synapse, to the somatic compartment^34^. Here we used a modified version of this method. We used Neuron_Reduce to generate a multi-cylindrical model of the cell as in Fig. 1b. Then, all the synapses in the original tree were mapped to the model soma. To account for dendritic filtering, for each synapse, we multiplied the original synaptic conductance, *g*_syn_, by the steady-state voltage attenuation factor from the original dendritic location, *j*, of the synapse to the soma. Specifically,
  12 $$g_{{\mathrm{syn}}}^ \ast = g_{{\mathrm{syn}}} \ast \frac{{| {Z_{0,j}} |}}{{| {Z_{0,0}} |}} = g_{{\mathrm{syn}}} \ast \frac{{V_{0,j}}}{{V_{0,0}}},$$
  where $g_{{\mathrm{syn}}}^ \ast$ is the new synaptic weight for synapse *j* when placed at the soma of the reduced model.

### Spatio-temporal patterns of synaptic activation

In Fig. 6, 12 synapses, placed at 25 µm intervals, were distributed on a stretch of one basal dendrite. The peak AMPA conductance per synapse was 5 nS. In cases where the synapses also had an NMDA component, the NMDA-based peak conductance was 3.55 nS. The synapses were activated in a specific temporal order with a time delay of 3.66 ms between them. This resulted in an input velocity of 7 µm/s for the sequential IN and OUT patterns in Fig. 6. In addition, the temporal order of synaptic activation was randomized and scored according to the directionality index^52^, which sums the number of swaps used by the bubble-sort algorithm to sort a specific temporal pattern into the IN pattern. In this measure, an IN pattern is attributed the value of 0 (no swaps) and the OUT pattern the value of 67 (67 swaps in bubble sort are required to “sort” the OUT pattern into the IN pattern^52^).

All simulations were performed using NEURON 7.4–7.7^20^ running on the Blue Brain V supercomputer based on HPE SGI 8600 platform hosted at the Swiss National Computing Center in Lugano, Switzerland. Each compute node was composed of an Intel Xeon 6140 CPUs @2.3 GHz and 384 GB DRAM. Analysis and simulation were conducted using Python and visualization using Matplotlib^66^.

The Neuron_Reduce algorithm is publicly available on GitHub (http://github.com/orena1/neuron_reduce).

### Reporting summary

Further information on research design is available in the Nature Research Reporting Summary linked to this article.

## Supplementary information

Supplementary Information

Peer Review File

Reporting Summary

## Data Availability

All spike times and somatic membrane potentials presented in the article are available upon request.

## Code availability

The Neuron_Reduce algorithm and most of the models that were used in the paper are publicly available on GitHub (http://github.com/orena1/neuron_reduce). An interactive example is available as a live paper (https://humanbrainproject.github.io/hbp-bsp-live-papers/2020/amsalem_et_al_2020/amsalem_et_al_2020.html). Software used for visualization of neurons in Fig. 7 is available at https://github.com/BlueBrain/RTNeuron.
