# Supplementary material for: An efficient analytical reduction of detailed nonlinear neuron models
Source: Nat Commun. 2020 Jan 15;11:288. doi: 10.1038/s41467-019-13932-6 (PMC6962154; doi:10.1038/s41467-019-13932-6)
Supplement: Supplementary file 1 — Supplementary Information [file 41467_2019_13932_MOESM1_ESM.pdf]

Supplementary Information

An efficient analytical reduction of detailed nonlinear neuron models

[1]Oren Amsalem*, [1]Guy Eyal, [1]Noa Rogozinski, [2]Michael Gevaert, [2]Pramod Kumbhar, [2]Felix Schürmann and [1,3]Idan Segev

[1]Department of Neurobiology, Hebrew University of Jerusalem, 9190401 Jerusalem, Israel. [2]Blue Brain Project, École Polytechnique Fédérale de Lausanne (EPFL), Campus Biotech, 1202 Geneva, Switzerland. [3]Edmond and Lily Safra Center for Brain Sciences, Hebrew University of Jerusalem, 9190401 Jerusalem, Israel.

* Correspondence: Oren Amsalem - oren.amsalem1@mail.huji.ac.il

Keywords: Neuron_Reduce, Compartmental modeling, Nonlinear dendrites, Cortical synapses, Single neuron computation, Cable theory

Supplementary Information

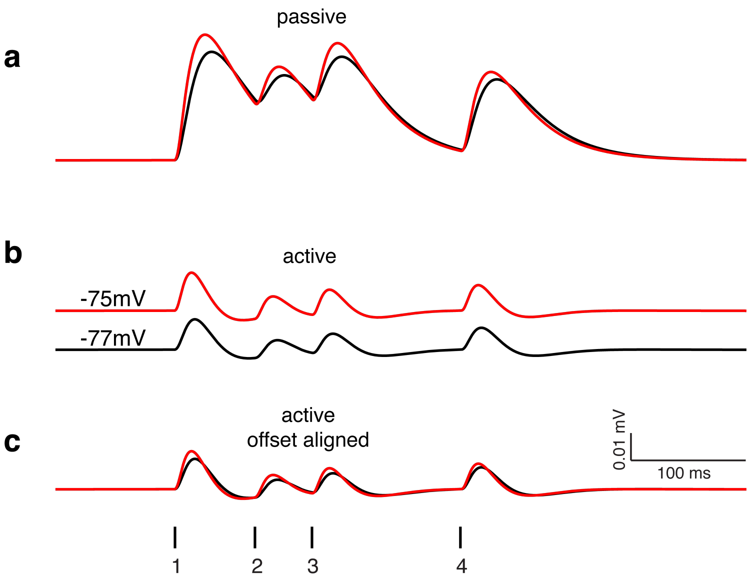

Supplementary Figure 1. Voltage response for synaptic activation in passive and active models of L5 pyramidal cells. **a** Sequential activation of the four synapses shown in **Fig. 1c** in a passive model of an L5 pyramidal cell (black) and its reduced model (red), as in **Fig. 1e**. **b** Activation of the same synapses, but on the detailed active models of this cell (similar colors). Note that in the active model, the resting potential in the reduced model is more depolarized than in the detailed model. **c** same as in b, but with the super-positioning of the resting potential in the two models.

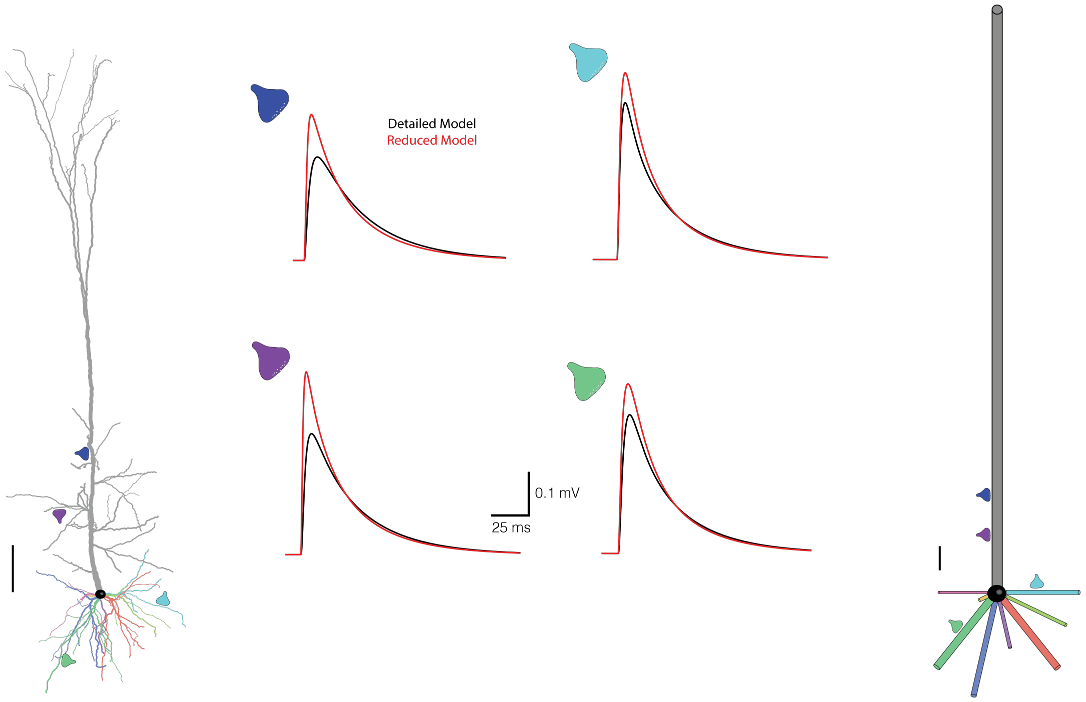

Supplementary Figure 2. Somatic voltage response in the detailed vs. the reduced model of a layer 5 pyramidal cell. Left and right, the detailed and reduced models as in **Fig. 1**. Middle, EPSPs measured at the soma of the detailed (black traces) and reduced (red traces) models for the four simulated synapses. Location of synapses is shown by respective colored synapses on the tree; their EPSPs are also shown by the respective colored synapses near the voltage traces. Note that as expected from **Fig. 1d**, at high frequencies (e.g., at the EPSP peak) the reduced model overestimated the peak value at the soma, but the

time-integrals of the EPSP in the two models remain similar (they are identical, in the passive case, since the time-integral behaves as in the steady-state case[1]). Synapse rise and decay times were 0.3 and 1.8 ms respectively, and the peak synaptic conductance was 0.8 nS. Scale bar is 100 µm in both the detailed and reduced models.

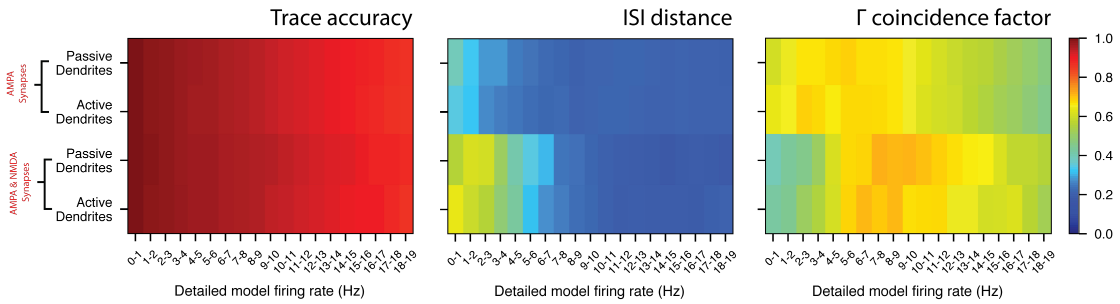

Supplementary Figure 3. *Neuron_Reduce* performance quantified using different synchronization metrics. The performance of *Neuron_Reduce* on the L5PC shown in **Fig. 2a** as compared to that of the detailed model as a function of the firing rate of the detailed model, for active and passive dendrites and with/without NMDA- based synaptic conductance (same conditions as in **Fig. 2g**). Three different quantification methods for the quality of *Neuron_Reduce* performance are shown: Trace accuracy[2], ISI distance[3], and the Γ coincidence factor[4]. In both the Trace accuracy and the Γ coincidence factor, higher values represent better synchronization while in the ISI distance method, lower values represent higher synchronization.

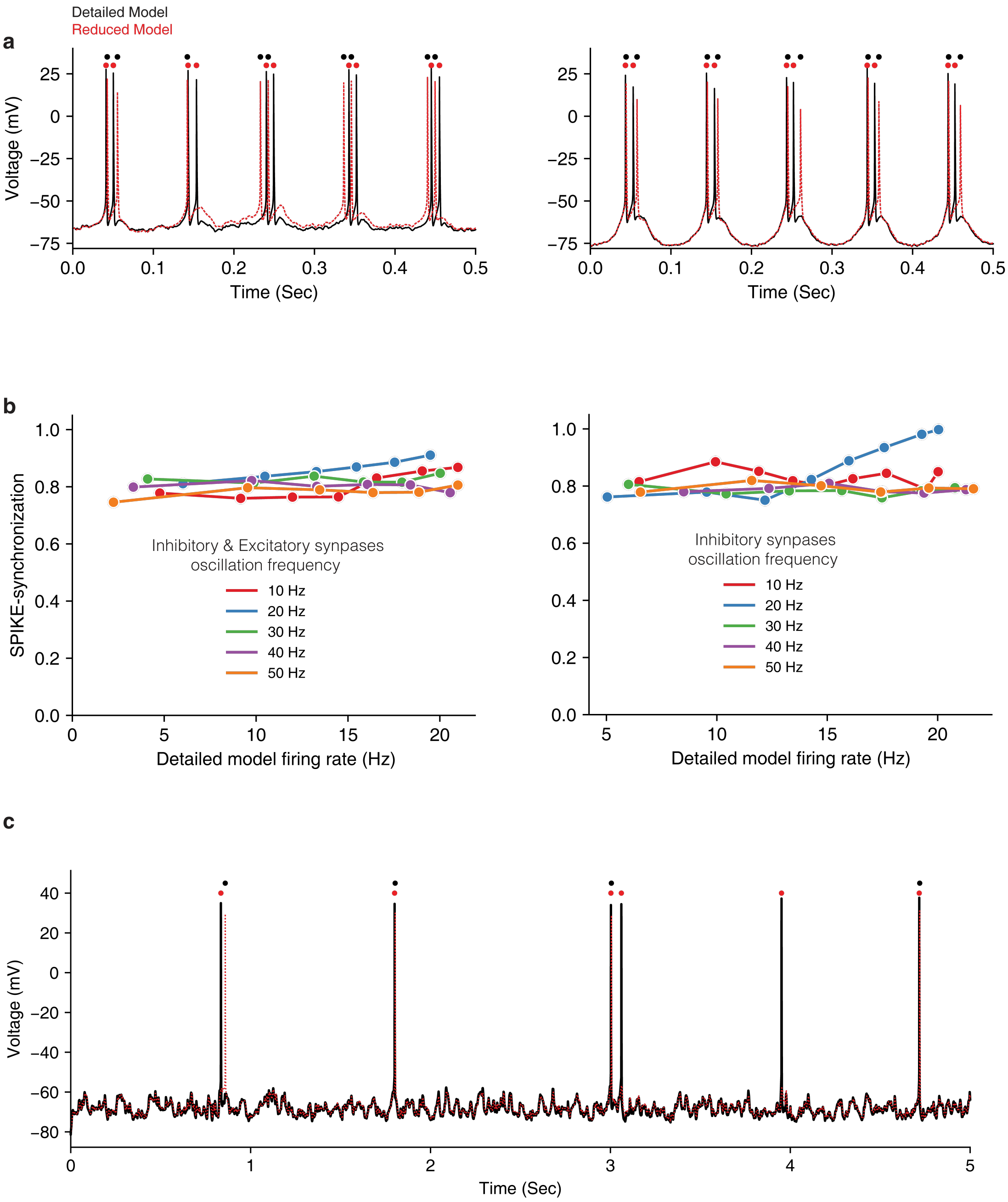

Supplementary Figure 4. ***Neuron_Reduce*** **performance under realistic synaptic input. a** Left, voltage dynamics at the soma of the detailed model (black trace) and the reduced model (red trace) of the L5PC model shown in **Fig. 2a.** The inhibitory and excitatory input was oscillating at 10 Hz; the excitatory synapses were activated at a rate of 6.7 Hz (see **Methods**). Right, Same as in left, but in this case only the inhibitory synapses were oscillating. **b** Left, SPIKE-synchronization as a function of the detailed model firing rate for different oscillating frequencies for the case where both excitatory and inhibitory synapses were oscillating. Right, as in left, but when only the inhibitory synapses were oscillating. **c** Voltage dynamics at the soma of the detailed model (black trace) and of the reduced model (red trace) of a Layer 5 Thick-tufted Pyramidal Cell with an early bifurcating apical tuft (L5_TTC2) extracted from active Blue Brain microcircuit[5] (see **Methods**). The synaptic activation from the microcircuit was replayed to the detailed model and the resultant somatic voltage is shown in black. The cell was simplified using *Neuron_Reduce* and the resultant voltage trace is shown in red. SPIKE-synchronization was of 0.71.

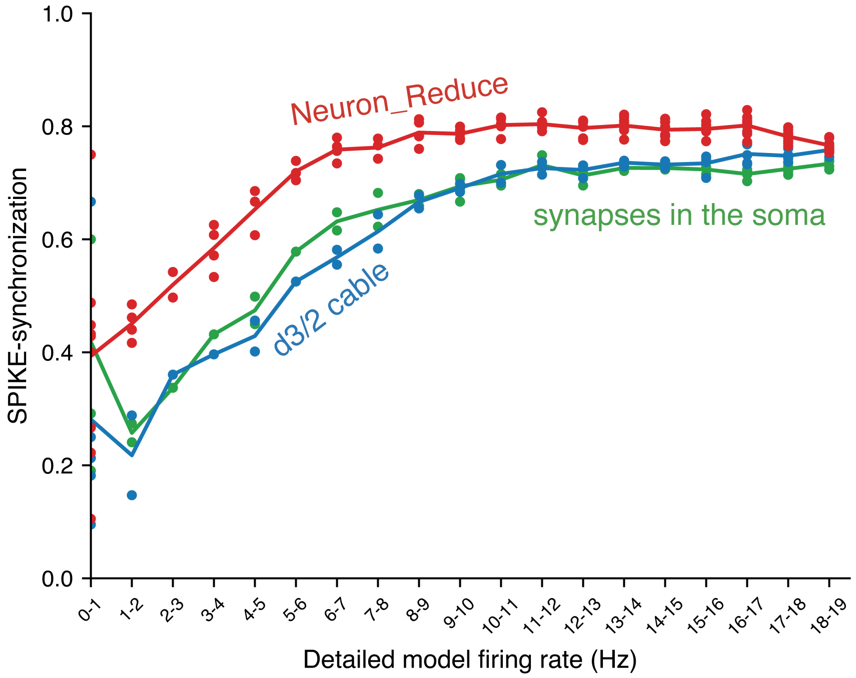

Supplementary Figure 5. **The accuracy of *Neuron_Reduce* compared to two other simplification methods.** Accuracy was measured via SPIKE-synchronization as a function of the firing rate of the detailed model. Three reduction methods are compared: *Neuron_Reduce* (red), Rall's d3/2 reduction method ("equivalent cable" approach, blue), and the mapping of all the synapses to the soma (green) see **Methods** for details on these reduction methods. Note that the accuracy of *Neuron_Reduce* is better than that of the other two methods across the entire range of model responses.

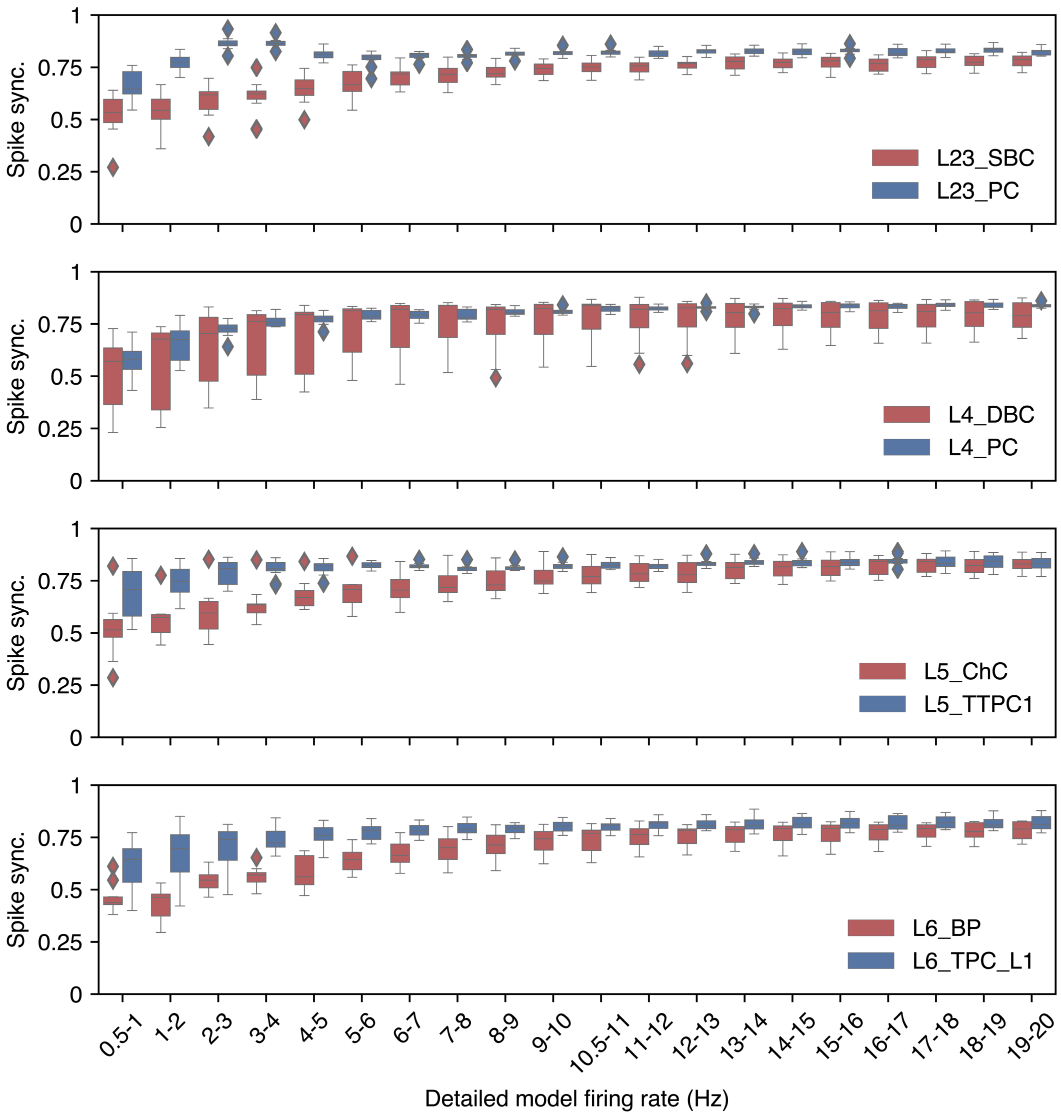

Supplementary Figure 6. **The accuracy of *Neuron_Reduce* on a large sample of neocortical neurons.** Boxplot of Spike-synchronization measured on 8 morphological types from the Blue Brain database[5,6]. 11 models were sampled for each type (88 cells in total), and simulations were executed for 30 seconds for each output frequency (x-axis). Each excitatory neuron received 10,000 synapses, and each inhibitory neuron received 3,000 synapses. Synaptic input for the excitatory neurons was as for tufted PC (L6) shown in **Supplementary Table 2** (5th row). Synaptic input for the inhibitory neurons was as for the L5 Martinotti cell shown in **Supplementary Table 2** (9th row). The number of compartments in the reduced model was set to 10% of the number of compartments in the detailed model when spatial discretization, ΔX, per compartment of 0.1λ resulted in a total number of compartments that is lower than 10% of the number of compartments in the detailed model (See **Supplementary Table 1**). SBC – Small Basket Cell; DBC – Double Bouquet Cell; ChC – Chandelier Cell; BP – Bipolar Cell; PC – Pyramidal Cell; TTPC1 – Thick-tufted Pyramidal Cell with a late bifurcating apical tuft; TPC_L1 – Tufted Pyramidal Cell with apical dendrites terminating in layer 1. Box bounds spans from 25 to 75% percentile, center line represents median, and whiskers extend to ± 1.5 interquartile range.

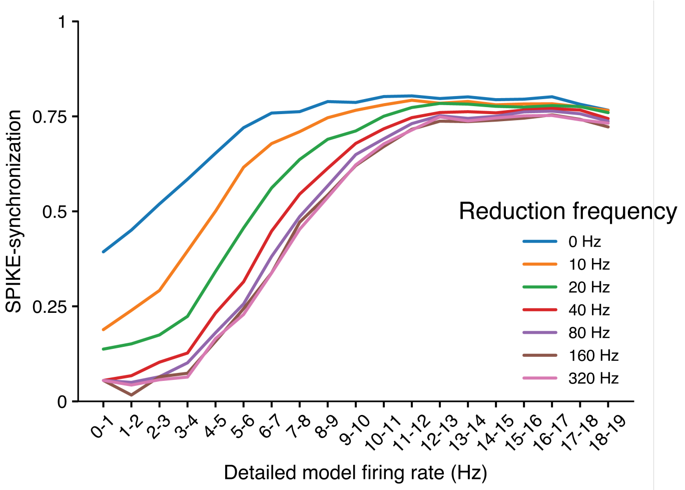

Supplementary Figure 7. **Reduction performance ω values.** Spike synchronization as a function of the spike firing in the detailed model for different frequencies, ω, used in Neuron_Reduce (Eqs. (1)-(11)). Model used as in **Fig. 2f** for the case denoted by the orange line. Note that the case of ω = 0 outperforms all other cases.

Reduction steps	Total run-time (s)	Run time speed-up factor	Number of compartments *(run-time, s)*	Number of synapses *(run-time, s)*
Detailed model	2,906	1	642 (*796.5*)	10,000 (*1,942.6*)
Morphological reduction	2,036	1.4	50 (*55.7*)	10,000 (*1,891.4*)
Synaptic merging	1005.5	2.9	642 (*762.8*)	1234 (*154.5*)
Neuron_Reduce	68.7	42	50 (*46.3*)	94 (*8.5*)

Supplementary Table 1. Enhanced simulation speeds in the reduced models versus the detailed models of a L5 pyramidal cell. The modeled cell is shown in **Fig. 1**; simulations were performed with 10,000 synapses; simulation time was 50 seconds, excitatory and inhibitory synapse firing rate was 5 and 10 Hz, respectively (see **Fig. 2** and **3).** Total run time for the detailed model is shown in the first row, first column of the shaded region. In the second row, the detailed dendritic morphology (consisting of 642 compartments) was mapped to its respective multipolar cylindrical representation consisting of only 50 compartments (as in **Fig. 1b**), whereas the total number of synaptic point processes (10,000) was kept as in the detailed model (1.39 folds reduction in run-time). In the third row, the detailed morphology remained untouched but synapses located on the same compartment were merged into a single point process (~2 point processes/compartment, one representing all E-synapses and the other all I-synapses located on this compartment). This resulted in 1234 synapses and a 2.9-fold reduction in run-time. In the bottom row, we used the full *Neuron_Reduce* algorithm, in which both the morphology reduction and the synaptic merging algorithms were implemented (50 compartments and 94 synaptic point processes), resulting in a 45-fold reduction in total simulation run-time (see also **Fig. 3a**). Columns 4 and 5 shed light on the underlying reasons for the speed-up: the runtime is dominated by two contributions, a) the number of compartments and b) the number of synapses. In brackets, we also list the resulting runtimes (Please note: since the total runtime has a third contribution from solving the linear system of the branched tree (not shown), the runtimes given in brackets do not add up to the total runtime). In the detailed model, the contributions from the compartments and synapses are 1/3 and 2/3, respectively. In the cases of only morphological reduction or only synaptic merging, the speed-up factor accordingly is limited by that contribution's weight in the sum. Only when reducing both at the same time, the full speed-up can be realized. The small runtime difference for 50 compartments in the morphological reduction vs. *Neuron_Reduce* case is attributed to more memory overhead in the case of the

morphological reduction with a much larger number of synapses. Note that these calculations are based on 10 repetitions for each condition, on a cluster with Intel Xeon 6140 CPUs and NEURON version 7.4 (see **Methods**). For each single cell, we used a single core on each node allocated exclusively to avoid side effects from other running processes.

model	Number of synapses	AMPA strength (nS)	NMDA strength (nS)	Inhibitory strength (nS)	AMPA decay time constant (ms)*	NMDA decay time constant (ms)**	GABA decay time constant (ms)***	Excitatory activation rates (Hz)	Temp (℃)	Used in Figures	Citations and links
L5PC model	10000	0.4	0.28	1	1.7	43	8	3 - 8	37	2-6,S3-S5,S7	[7] modelDB accession number 139653
L5PC no NMDA	10000	0.4	-	1	1.7	-	8	8 - 20	37	1,2,5,S1,S3	[7] modelDB accession number 139653
L5PC passive dends	10000	0.4	0.28	1	1.7	43	8	3 - 8	37	2,S3	[7] modelDB accession number 139653
L5PC passive dends no NMDA	10000	0.4	-	1	1.7	-	8	8 - 20	37	1,2,S1,S2,S3	[7] modelDB accession number 139653
Tufted PC (L6)	10000	0.79	0.56	0.88	1.74	43	7.65	1 - 3	34	7	[5] https://bbp.epfl.ch/nmc-portal/documents/10184/1921826/L6_TPC_L1_cADpyr231_5.zip
Large Basket Cell (L2/3)	1250	0.69	-	0.125	2	-	8	1 - 4	34	7	[8]
Double Bouquet Cell (L4)	2000	0.41	0.29	0.84	1.73	43	8.32	2 - 4	34	7	[5] https://bbp.epfl.ch/nmc-portal/documents/10184/52145/L4_DBC_cNAC187_1.zip
Spiny Stellate Cell (L4)	5000	0.76	0.54	0.84	1.74	43	7.82	1 - 4	34	7	[5] https://bbp.epfl.ch/nmc-portal/documents/10184/1921834/L4_SS_cADpyr230_1.zip
Martinotti Cell (L5)	5000	0.12	0.085	0.84	1.74	43	8.34	7 - 19	34	7	[5] https://bbp.epfl.ch/nmc-portal/documents/10184/52298/L5_MC_bAC217_2.zip
Spiny Rbp4-Negative (L4)	5000	0.4	0.28	1	1.7	43	8	3 - 5	34	7	[9] http://celltypes.brain-map.org/neuronal_model/download/483108201
Aspiny - Htr3a-Positive (L1)	5000	0.4	-	1	1.7	-	8	6 - 10	34	7	[9] http://celltypes.brain-map.org/neuronal_model/download/478045081
Human Pyramidal Cell (L2/3)	10000	0.7	-	0.7	1.8	-	8	2 – 3	37	7	[10] https://senselab.med.yale.edu/ModelDB/showmodel.cshtml?model=195667
Thalamocortical cAD	2000	0.4	0.28	1	1.7	43	8	4-9	34	7	[11]https://senselab.med.yale.edu/modeldb/ShowModel.cshtml?model=251881
Thalamocortical cNAD	2000	0.4	0.28	1	1.7	43	8	4-9	34	7	[11]https://senselab.med.yale.edu/modeldb/ShowModel.cshtml?model=251881
Basal ganglia medium spiny neuron	5000	0.15	0.15	0.45	1.7	43	8	5-7	35	7	[12]https://humanbrainproject.github.io/hbp-bsp-live-papers/2018/lindroos_et_al_2018/lindroos_et_al_2018.html
Cerebellar Golgi cell	5000	0.4	0.28	1	1.7	43	8	4-5	23	7	Egidio D'Angelo's Lab (Personal communication)
Hippocampus inhibitory neuron	7000	0.4	0.28	1	1.7	43	8	4-12	34	7	[13]modelDB accession number 244688; cell_seed2_0-cnac_08

Supplementary Table 2. Neuron models and synaptic parameters used in this article

* the rise time constant for the AMPA synapses was 0.2 ms in all models except the Large Basket Cell (0.3 ms) and the Human Pyramidal Cell (0.3 ms)

** the rise time constant for the NMDA synapses was 0.29 ms in all models with NMDA synapses

**** the rise time constant for the GABA synapses was 0.2 ms in all models except the Large Basket Cell (1 ms)

Supplementary References

1. Rinzel, J. & Rall, W. Transient response in a dendritic neuron model for current injected at one branch. *Biophys. J.* **14**, 759–790 (1974).
2. Marasco, A., Limongiello, A. & Migliore, M. Fast and accurate low-dimensional reduction of biophysically detailed neuron models. *Sci. Rep.* **2**, 928 (2012).
3. Kreuz, T., Mulansky, M. & Bozanic, N. SPIKY: a graphical user interface for monitoring spike train synchrony. *J. Neurophysiol.* **113**, 3432–3445 (2015).
4. Jolivet, R., Lewis, T. J. & Gerstner, W. Generalized Integrate-and-Fire Models of Neuronal Activity Approximate Spike Trains of a Detailed Model to a High Degree of Accuracy. *J. Neurophysiol.* **92**, (2004).
5. Markram, H. *et al.* Reconstruction and Simulation of Neocortical Microcircuitry. *Cell* **163**, 456–92 (2015).
6. Ramaswamy, S. *et al.* The neocortical microcircuit collaboration portal: a resource for rat somatosensory cortex. *Front. Neural Circuits* **9**, 44 (2015).
7. Hay, E., Hill, S., Schürmann, F., Markram, H. & Segev, I. Models of neocortical layer 5b pyramidal cells capturing a wide range of dendritic and perisomatic active properties. *PLoS Comput. Biol.* **7**, e1002107 (2011).
8. Amsalem, O., Van Geit, W., Muller, E., Markram, H. & Segev, I. From Neuron Biophysics to Orientation Selectivity in Electrically Coupled Networks of Neocortical L2/3 Large Basket Cells. *Cereb. Cortex* **26**, 3655–3668 (2016).
9. Gouwens, N. W. *et al.* Systematic generation of biophysically detailed models for diverse cortical neuron types. *Nat. Commun.* **9**, 710 (2018).
10. Eyal, G. *et al.* Unique membrane properties and enhanced signal processing in human neocortical neurons. *Elife* **5**, e16553 (2016).
11. Iavarone, E. *et al.* Experimentally-constrained biophysical models of tonic and burst firing modes in thalamocortical neurons. *PLoS Comput. Biol.* (2019). doi:10.1371/journal.pcbi.1006753
12. Lindroos, R. *et al.* Basal Ganglia Neuromodulation Over Multiple Temporal and Structural Scales—Simulations of Direct Pathway MSNs Investigate the Fast Onset of Dopaminergic Effects and Predict the Role of Kv4.2. *Front. Neural Circuits* (2018). doi:10.3389/fncir.2018.00003
13. Migliore, R. *et al.* The physiological variability of channel density in hippocampal CA1 pyramidal cells and interneurons explored using a unified data-driven modeling workflow. *PLoS Comput. Biol.* (2018). doi:10.1371/journal.pcbi.1006423
