# Supplementary material for: An efficient analytical reduction of detailed nonlinear neuron models
Source: Nat Commun. 2020 Jan 15;11:288. doi: 10.1038/s41467-019-13932-6 (PMC6962154; doi:10.1038/s41467-019-13932-6)
Supplement: Supplementary file 2 — Peer Review File [file 41467_2019_13932_MOESM2_ESM.pdf]

Reviewers' comments:

Reviewer #1 (Remarks to the Author):

The manuscript by Amsalem and colleagues describes a new method and corresponding software, termed Neuron_Reduce, for systematic coarse-graining of morphologically detailed, compartmental neuronal models. Simulations of compartmental neuron models are computationally demanding, and reducing the computational expense of such simulations while preserving model accuracy is an important goal. This goal is becoming particularly crucial in recent years, as the field is moving towards large and complex network simulations of brain circuits, consisting of tens or hundreds of thousands of compartmental neuronal models, and beyond. Methods for systematic reduction of these network models, which require huge compute resources, to something that can be simulated for a fraction of the cost, will be a big help to the field.

The authors make substantial progress in this direction through the Neuron_Reduce method, which at this stage works at the level of individual neuronal models. They show that coarse-grained neuron models can be simulated 50-250 times faster than the original full models. They demonstrate that synaptic activation results in similar patterns of membrane voltage dynamics at the soma (and dendrites) between the coarse-grained and original model, including recapitulation of nonlinear phenomena such as the backpropagation of action potentials and dendritic calcium spikes. The spiking output of several neuronal models is shown to be mostly reproduced by coarse-grained versions of these models. These all are very positive results.

Overall, I find this study quite promising and exciting. The authors achieved impressive results in both accuracy and computational improvements. I congratulate them on this great work. I believe it will be of high interest to the community. It is possible that Neuron_Reduce will be widely used and will substantially speed up the progress in computational neuroscience.

However, I do see important caveats in the current version of the manuscript and urge the authors to address them. Two major issues are concerning (please see details below) – the very narrow testing of the method on a small number of cells and no investigation of neuronal responses in the context of realistic network dynamics. Fixing these two issues should not be too difficult and will result in an excellent and very convincing paper, but without them one remains wondering about broad utility of the methods.

Major issues.

1. Small number of neurons tested.

The authors show results for only one model, the active-dendrites model of a Layer 5 pyramidal cell of Hay et al., in 5 out of 6 figures. Fig. 6 shows results for 8 neurons. In this day and age, I think this is insufficient. Some of the authors are from the Blue Brain Project, which has models of hundreds or thousands of neurons. I think results for a large number of such models should be analyzed and presented in a systematic way. Alternatively, one could analyze Neuron_Reduce's coarse-graining performance on a large number of models from the Allen Institute's Cell Types database, which contains models of hundreds of neurons from the mouse and human cortical tissue. Finally, as far as I know, both of these sources contain mostly cortical neurons, and it would be important to demonstrate utility for other types of neurons as well. Those can be found in the ModelDB database. Of course, it would be too hard to study all possible neurons from ModelDB, but showing how Neuron_Reduce works for 10-20 non-cortical and/or non-mammalian neurons would be very helpful.

I therefore encourage the authors to perform analysis of hundreds of diverse neurons and present statistics of the results. Ideally, that would include synaptic activations, but at the very least it would be helpful to explore performance on current injections. The latter should be particularly easy, given

that current injections into the full neuron models should be available for both Blue Brain and Allen Institute databases.

In terms of reporting the results, one could use something like Fig. 6G (obviously, for many more neurons), where I would recommend sorting the neurons according to their performance. Ideally, one would also group neurons into biological types and present statistics (box plots or distributions) of various measures for each group. The measures should include the SPIKE-synchronization index as well as others shown in Fig. S3, but I also would recommend checking metrics like rheobase, f-I curve slope, action potential width and amplitude, burstiness, etc.

2. No testing of performance under realistic conditions of synaptic inputs from an active network.

The most important application of this work is likely going to be simplification of network simulations. However, the authors only investigate synaptic activations using random spike trains, which likely do not represent very well the structured inputs received by neurons in the context of biological brain networks. In fact, the authors favor the omega=0 case, but neuronsin brain networks receive inputs that are often well synchronized at certain frequencies, such as alpha, theta, gamma, and others – from sub-1 Hz to tens of Hz. These frequencies are ubiquitous and may be critical for brain function. Therefore, one wonders how the omega=0 case would work for more realistic inputs that contain strongly oscillating components at diverse frequencies.

I think the authors could do such tests with reasonably limited effort. Wang et al. (Science, 2010, doi: 10.1126/science.1183108) used very successfully the approach where experimentally recorded spike trains were used to activate a cortical neuron. Alternatively, again, the Blue Brain Project has network simulations that were published. One could take spike trains from these simulations converging on single neurons, and test what responses such spike trains would elicit in coarse-grained versions of these neurons. Likewise, the Allen Institute has made their network model of the Layer 4 of mouse cortex publicly available. Spike trains from that model could be used as well. In fact, spike trains from either or both of these studies could be used for many different neurons, not necessarily exactly those from the original networks. The point is simply that spike trains from these network simulations are likely more representative of biologically relevant activation patterns than what is provided by random spike trains.

I think performing such tests is crucial. One reason is that Figs. 2G and 6G show that for several of the tested neurons the SPIKE-synchronization measure is only ~0.5 or even below for low firing frequencies. This is very concerning. Although it's great that the performance improves for firing rates >5 Hz, those rates are relatively rare for cortical pyramidal neurons. Frequencies of 5 Hz or above are typically observed when neurons are stimulated with their most preferred stimuli, whereas for all other conditions these neurons are usually much less active; thus, they spend most of the time in real awake brain firing at frequencies well below 5 Hz. It seems, therefore, that a network model consisting of reduced neuron models would perform quite poorly for most of neurons in the network most of the time.

Thus, it is essential to investigate performance of a large number of diverse neurons under realistic spike trains to judge how well the Neuron_Reduce method works for biologically relevant situations. Ideally, one would actually perform coarse-grained network simulations, but I see the authors' point that perhaps these can be addressed in subsequent work.

Minor issues.

1. The authors show some comparisons with other coarse-graining methods, but it would be good to see more. In particular, they show that Neuron_Reduce provides certain preservation of accuracy and certain speed-up, but how do those results compare with what one would achieve using radically

simpler methods? I would recommend using the simplest leaky integrate-and-fire (LIF) model for each neuron tested, and perhaps also more complex LIF model, like the one from Pozzorini et al. (PLoS Comp. Bio., 2015). I assume that speed-up of those methods will be better than that of Neuron_Reduce, but perhaps the accuracy won't be as good.

2. Page 3, second paragraph. It may be worth adding references in the following places:

"simulate large parts of the visual system of the mouse" – as noted above, the Allen Institute recently published their network model of Layer 4 (Arkhipov et al., PLOS Comp. Bio., 2018).

"computer software that implements detailed CMs" – perhaps also add NetPyNE (Dura-Bernal et al., BioRxiv, doi: https://doi.org/10.1101/461137), Allen Institute's Brain Modeling ToolKit (Gratiy et al., PLOS ONE, 2018), and PyNN (Davison et al., Front. Neuroinform., 2009).

3. Page 5, last paragraph: "Conveniently, we found a close match between the detailed and the reduced models for omega=0 (the steady-state case); this is depicted in Figure 1D and Figure S1" – I don't think this is shown in Fig. S1. The Methods says "data not shown", but I think this is important data that should be shown.

4. Page 6, second paragraph: "In the reduced model, these synapses are merged into one NEURON process (red synapse in Figure 1B). However, they retain their individual activation times (see Methods)." For what it's worth, I am concerned about how well this will scale for parallelized network simulations. I understand that this is not in the scope of the current manuscript, but the authors may want to clarify what one should expect. Also, as the authors point out in a different part of the manuscript, this scheme currently does not support merging synapses with different kinetics parameters, which may prevent full utilization of synapse merging benefits for large heterogeneous network simulations.

5. Page 9, top: "We also tested other spike trains similarity metrics (Figure S3) and found comparable results to those shown in Figure 3." Should this refer to Figure 2 instead?

6. Page 22, third paragraph: "All synapses with a similar |Z_(0,x) (omega)| value…" – what does "similar" mean? Are they similar within some tolerance, or were they somehow binned?

7. References: Ref. 45 is a duplication of Ref. 30; Ref. 56 is a duplication of Ref. 17.

Reviewer #2 (Remarks to the Author):

This study reports a systematic way to reduce complicated neuronal models.
This approach is extremely interesting in principle to many modelers,
as the computation time for detailed compartmental neuronal models is
difficult to reduce even through parallelization.
While I am entirely in favor of the objectives, I feel that the approach needs
to be better characterized, and there are some use cases that should be better
explored and reported.

In my reading of the paper, I considered four specific challenges for the
proposed method:
1. How does the response look at the soma?
Relevant for I/O relationships.
2. How does the local dendritic response look?

Relevant for such processes as dendritic plasticity,
local summation and AP generation
3. How is the coupling with the soma?
Dendritic APs -> somatic responses.
BPAPs
4. How does intracellular chemical signaling work if the neuron is subdivided with this approach?
Relevant for local plasticity, synaptic tagging, and structural plasticity processes.

In order:

1. How does the response look at the soma?
This is pretty good. The authors did look at random input throughout the cell. However, I would like to see two additional tests:
1.1: an EI balance test, considering whether local EI balance gives the same result in the reduced model.
1.2: Rhythmic activity imposed by periodic inhibitory input on top of steady random background input. Does the somatic firing and impedance behave the same in full vs. reduced models?

2. How does the local dendritic response look?
2.1 This was tested for Rall's and Hausser's directionality detection. It is good to see that it works for these cases.
2.2 I would like to see it tested for local (dendritic) spiking. Could the authors deliver random input with a certain distribution on the synapses, and report on triggering of local dendritic spikes?
2.3 The authors briefly report differences with local potentials, it would be good to have this summarized and charted for the different cases of a Ca spike on an apical dendrite, and for different frequencies of local dendritic synaptic input.
2.4 I would like to see what happens to dendritic potentials for random cell-wide input. Figs 2 and 6 don't really show this.
2.5 As the authors have themselves discussed in previous work, an interesting proposed computation in the cell is to have geometrically defined local inhibition, such as shunt inhibition, on one or other side of a dendritic branch.
How does this carry over to the reduced model?
2.6 What would happen if there were dendro-dendritic or gap-junction input to dendrites? Does this reduce cleanly or poorly?

3. How is the bidirectional coupling with the soma?
3.1 Dendritic APs -> somatic potentials.
This was done for a single dendritic Ca spike (Figure 4) but not for other forms of local dendritic activity.
3.2 BPAPs
Tested. This looks reasonable.
4. Can this mapping preserve intracellular chemical as well as electrical signaling, in local dendritic domains ?
4.1 I understand that the authors have focused on electrical properties, but I would like to see at least a considered discussion on how model reduction affects this aspect

of cellular signaling. For example, collapsing all synapses of a dendrite into a single synapse would seem to completely eliminate all possibility of synaptic tagging or biochemical sequence computation, not to mention reducing potential network memory capacity by orders of magnitude.
4.2 There is considerable evidence for local dendritic CICR (e.g., Plotkin's work). How could this map? I would like to see simulations.

Beyond these specific challenges, and as a further test of the approach, here are a couple of suggestions:
5. Introduce a reduced cell as a replacement for a detailed cell in a regular Blue-brain type cortical simulation. Ask how its spiking output compares with the output of the original cell.

6. In the same cell, ask if the synapses obeyed STDP rules, would they change in the same way? The authors should compare with original detailed model.

7. I also have a technical concern about the stated speedup with respect to synapses. In very large network simulations, much of the computation time is actually spent on synaptic transmission. This is even more so in the case of plastic synapses.
While it may be true that this method gives a 10-100 fold speedup for the cell model alone, it wasn't clear to me how the collapsed synapses were handled and how this scaled.
7.1 For example, if you have 100 inputs coming onto a single synaptic point process on a compartment, are the weights still changing independently?
7.2 I would like to see a test of this proposed speedup for a network. My impression is that more than half the computation time may be spent in transmission and synaptic rules, so all that this method could do would be to improve the runtime by 50% or so, and possibly much less. This should be explicitly measured for simple and for plastic synapses. My sense is that the shared synaptic point process may not be the major term in the computational cost for large numbers of synapses.

Overall, I think that the method is potentially very interesting. However, for the stated goal of the authors (to reduce highly detailed cell models for use in large network simulations) I feel that the current analysis doesn't go far enough. Specifically, there are a number of likely dendritic input scenarios in which I suspect the approach may provide degraded accuracy. These should be well characterized and systematically reported (e.g., in a table) for readers to be better able to decide when to use such a model reduction approach. I also have a technical concern about whether the reported speedup really holds in a network full of plastic synapses.

Reviewers' comments:

Reviewer #1 (Remarks to the Author):

The manuscript by Amsalem and colleagues describes a new method and corresponding software, termed Neuron_Reduce, for systematic coarse-graining of morphologically detailed, compartmental neuronal models. Simulations of compartmental neuron models are computationally demanding, and reducing the computational expense of such simulations while preserving model accuracy is an important goal. This goal is becoming particularly crucial in recent years, as the field is moving towards large and complex network simulations of brain circuits, consisting of tens or hundreds of thousands of compartmental neuronal models, and beyond. Methods for systematic reduction of these network models, which require huge compute resources, to something that can be simulated for a fraction of the cost, will be a big help to the field.

The authors make substantial progress in this direction through the Neuron_Reduce method, which at this stage works at the level of individual neuronal models. They show that coarse-grained neuron models can be simulated 50-250 times faster than the original full models. They demonstrate that synaptic activation results in similar patterns of membrane voltage dynamics at the soma (and dendrites) between the coarse-grained and original model, including recapitulation of nonlinear phenomena such as the backpropagation of action potentials and dendritic calcium spikes. The spiking output of several neuronal models is shown to be mostly reproduced by coarse-grained versions of these models. These all are very positive results.

Overall, I find this study quite promising and exciting. The authors achieved impressive results in both accuracy and computational improvements. I congratulate them on this great work. I believe it will be of high interest to the community. It is possible that Neuron_Reduce will be widely used and will substantially speed up the progress in computational neuroscience.

However, I do see important caveats in the current version of the manuscript and urge the authors to address them. Two major issues are concerning (please see details below) – the very narrow testing of the method on a small number of cells and no investigation of neuronal responses in the context of realistic network dynamics. Fixing these two issues should not be too difficult and will result in an excellent and very convincing paper, but without them one remains wondering about broad utility of the methods.

Major issues.

1. Small number of neurons tested.

The authors show results for only one model, the active-dendrites model of a Layer 5 pyramidal cell of Hay et al., in 5 out of 6 figures. Fig. 6 shows results for 8 neurons. In this day and age, I think this is insufficient. Some of the authors are from the Blue Brain Project, which has models of hundreds or thousands of neurons. I think results for a large number of such models should be analyzed and presented in a systematic way. Alternatively, one could analyze Neuron_Reduce's coarse-graining performance on a large number of models from the Allen Institute's Cell Types database, which contains models of hundreds of neurons from the mouse and human cortical tissue. Finally, as far as I know, both of these sources contain mostly cortical neurons, and it would be important to demonstrate utility for other types of neurons as well. Those can be found in the ModelDB database. Of course, it would be too hard to study all possible neurons from ModelDB, but showing how Neuron_Reduce works for 10-20 non-cortical and/or non-mammalian neurons would be very helpful.

I therefore encourage the authors to perform analysis of hundreds of diverse neurons and present statistics of the results. Ideally, that would include synaptic activations, but at the very least it would be helpful to explore performance on current injections. The latter should be particularly easy, given that current injections into the full neuron models should be available for both Blue Brain and Allen Institute databases.

In terms of reporting the results, one could use something like Fig. 6G (obviously, for many more neurons), where I would recommend sorting the neurons according to their performance. Ideally, one would also group neurons into biological types and present statistics (box plots or distributions) of various measures for each group. The measures should include the SPIKE-synchronization index as well as others shown in Fig. S3, but I also would recommend checking metrics like rheobase, f-I curve slope, action potential width and amplitude, burstiness, etc.

We thank the referee for his/her comment. Indeed, we quantified the performance of *Neuron_Reduce* only on neocortical neurons. Following the referee's request, we have performed the spike-synchrony analysis on additional 5 cells types (but not on "hundreds of cells") from a variety of brain regions and cell types. These include: Thalamic neurons, cerebellar Golgi cell, medium spiny neuron from the basal ganglia and Hippocampus inhibitory neuron. In the revised version, these results were added to the original Figure 6G (Now Figure 7G, see below). We did not, however, find good cell models from non-mammalian to test *Neuron_Reduce*. Importantly, in all modeled cases, the spike-synchrony analysis was similar to that presented in the original Figure 6 (Now Figure 7).

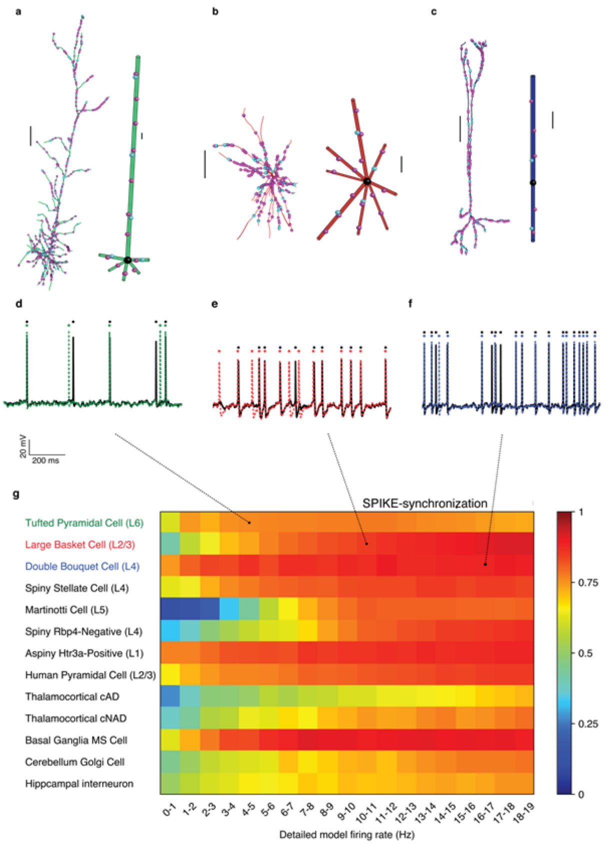

2. No testing of performance under realistic conditions of synaptic inputs from an active network.

The most important application of this work is likely going to be simplification of network simulations. However, the authors only investigate synaptic activations using random spike trains, which likely do not represent very well the structured inputs received by neurons in the context of biological brain networks. In fact, the authors favor the omega=0 case, but neurons in brain networks receive inputs that are often well synchronized at certain frequencies, such as alpha, theta, gamma, and others – from sub-1 Hz to tens of Hz. These frequencies are ubiquitous and may be critical for brain function. Therefore, one wonders how the omega=0 case would work for more realistic inputs that contain strongly oscillating components at diverse frequencies.

I think the authors could do such tests with reasonably limited effort. Wang et al. (Science, 2010, doi: 10.1126/science.1183108) used very successfully the approach where experimentally recorded spike trains were used to activate a cortical neuron. Alternatively, again, the Blue Brain Project has network simulations that were published. One could take spike trains from these simulations converging on single neurons, and test what responses such spike trains would elicit in coarse-grained versions of these neurons. Likewise, the Allen Institute has made their network model of the Layer 4 of mouse cortex publicly available. Spike trains from that model could be used as well. In fact, spike trains from either or both of these studies could be used for many different neurons, not necessarily exactly those from the original networks. The point is simply that spike trains from these network simulations are likely more representative of biologically relevant activation patterns than what is provided by random spike trains.

I think performing such tests is crucial. One reason is that Figs. 2G and 6G show that for several of the tested neurons the SPIKE-synchronization measure is only ~0.5 or even below for low firing frequencies. This is very concerning. Although it's great that the performance improves for firing rates >5 Hz, those rates are relatively rare for cortical pyramidal neurons. Frequencies of 5 Hz or above are typically observed when neurons are stimulated with their most preferred stimuli, whereas for all other conditions these neurons are usually much less active; thus, they spend most of the time in real awake brain firing at frequencies well below 5 Hz. It seems, therefore, that a network model consisting of reduced neuron models would perform quite poorly for most of neurons in the network most of the time.

Thus, it is essential to investigate performance of a large number of diverse neurons under realistic spike trains to judge how well the Neuron_Reduce method works for biologically relevant situations. Ideally, one would actually perform coarse-grained network simulations, but I see the authors' point that perhaps these can be addressed in subsequent work.

We thank the referee for this important point. as suggested by the referee we now extracted a Layer 5 Thick-tufted Pyramidal Cell with an early bifurcating apical tuft (L5_TTC2) from an active Blue Brain circuit simulation (whole-column consisting of ~30,000 interacting neurons, see (Markram et al., 2015)). This cell receives 4,984 synapses from this circuit. As suggested by the referee we replayed the synaptic activity impinging on this cell once on the detailed neuron model and once on the respective reduced model. In the figure constructed for the referee (Frame **c** in Figure below) we show the voltage response (5 sec, see **Methods**) of the detailed versus the reduced models. The Spike-synchronization in this case was 0.708, similar to the value we received for random inputs.

As suggested by the referee we also activated the synaptic input in an oscillatory manner (the activation of synapses was simulation as inhomogeneous Poisson process with a time-dependent intensity $\lambda(t) = \sin(t)$ see **Methods**), with spike synchronization of 0.78 in this case. We note that, even in this case, the best performance was achieved when we reduced the cell using omega = 0. The revise text now reads as follows:

We have also analyzed the performance of *Neuron_Reduce* on two additional patterns of synaptic input. In one case, the synaptic input was activated in an oscillatory manner at different frequencies (see **Methods**). In these cases, the spike synchronization measure ranged between 0.75 - 1 (**Supplementary Fig. 4a, b**). In the other case the synaptic input was taken from a spontaneously active Blue Brain circuit (Markram et al., 2015) (see **Methods**). In this case the spike synchronization measure was 0.708 (**Supplementary Fig. 4c**).

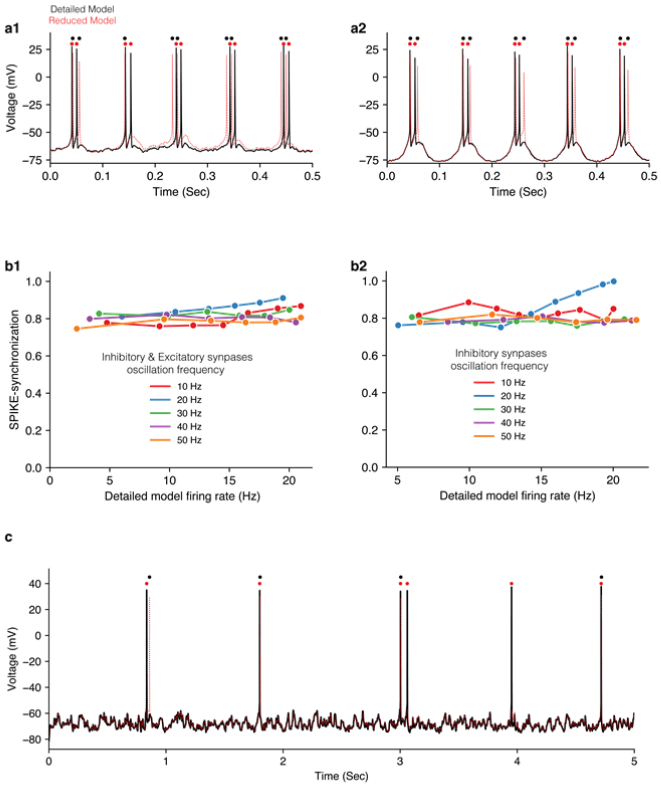

Minor issues.

1. The authors show some comparisons with other coarse-graining methods, but it would be good to see more. In particular, they show that Neuron_Reduce provides certain preservation of accuracy and certain speed-up, but how do those results compare with what one would achieve using radically simpler methods? I would recommend using the simplest leaky integrate-and-fire (LIF) model for each neuron tested, and perhaps also

more complex LIF model, like the one from Pozzorini et al. (PLoS Comp. Bio., 2015). I assume that speed-up of those methods will be better than that of Neuron_Reduce, but perhaps the accuracy won't be as good.

We thank the referee for this suggestion. We note that the aim of this work is to "preserve" dendrites and some of their filtering/nonlinear effects. W therefore did not systematically compare the performance of *Neuron_Reduce* to a "point neuron". Still, we did compare in **Supplementary Figure 5.** *Neuron_Reduce* performance to that where all the synapses are mapped to the soma of the modelled neuron while scaling their weights accordingly. The spike synchronization measure is worse in this case. We did not compare the speedup in this case. However, in this case all excitatory synapses could be merged into one "point process" and all inhibitory synapses to a second "point process". We thus expect a significant speedup for this "point neuron" case.

2. Page 3, second paragraph. It may be worth adding references in the following places: "simulate large parts of the visual system of the mouse" – as noted above, the Allen Institute recently published their network model of Layer 4 (Arkhipov et al., PLOS Comp. Bio., 2018).

Added

"computer software that implements detailed CMs" – perhaps also add NetPyNE (Dura-Bernal et al., BioRxiv, doi: https://doi.org/10.1101/461137), Allen Institute's Brain Modeling ToolKit (Gratiy et al., PLOS ONE, 2018), and PyNN (Davison et al., Front. Neuroinform., 2009).

Added

3. Page 5, last paragraph: "Conveniently, we found a close match between the detailed and the reduced models for omega = 0 (the steady-state case); this is depicted in Figure 1D and Figure S1" – I don't think this is shown in Fig. S1. The Methods says "data not shown", but I think this is important data that should be shown.

We thank the referee for this suggestion and, consequently, we compared in **Supplementary Figure 6** (see below) the quality of reduction for different omega values. As can be seen, the case of omega = 0 outperformed the other cases. This figure is now referred to in the **Discussion**.

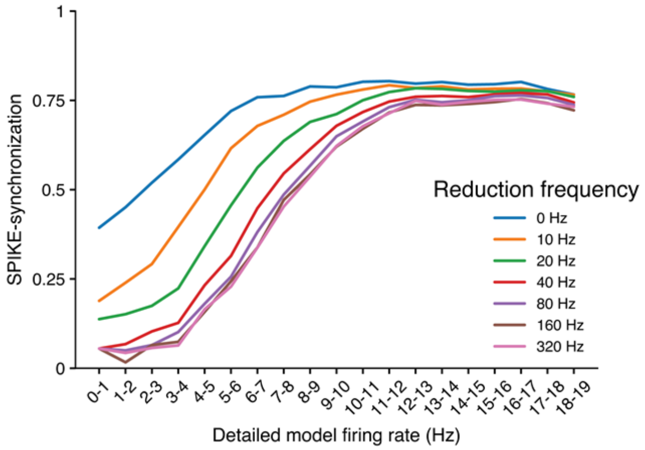

4. Page 6, second paragraph: "In the reduced model, these synapses are merged into one NEURON process (red synapse in Figure 1B). However, they retain their individual activation times (see Methods)." For what it's worth, I am concerned about how well this will scale for parallelized network simulations. I understand that this is not in the scope of the current manuscript, but the authors may want to clarify what one should expect. Also, as the authors point out in a different part of the manuscript, this scheme currently does not support merging synapses with different kinetics parameters, which may prevent full utilization of synapse merging benefits for large heterogeneous network simulations.

We thank the referee for these comments. Regarding the scaling to parallelized network simulations. We note that, there is no problem in keeping the individual activation time of each synapse when merged, also in parallel implementation of network simulation. For the referee, we added below two schematic figures to better explain the above claim.

Regarding merging synapses with different kinetics. This issue could be handled as we handled the case of excitatory versus inhibitory synapses. If there are more than one kinetic type of (say excitatory) synapses – then each type will be merged to a single point process.

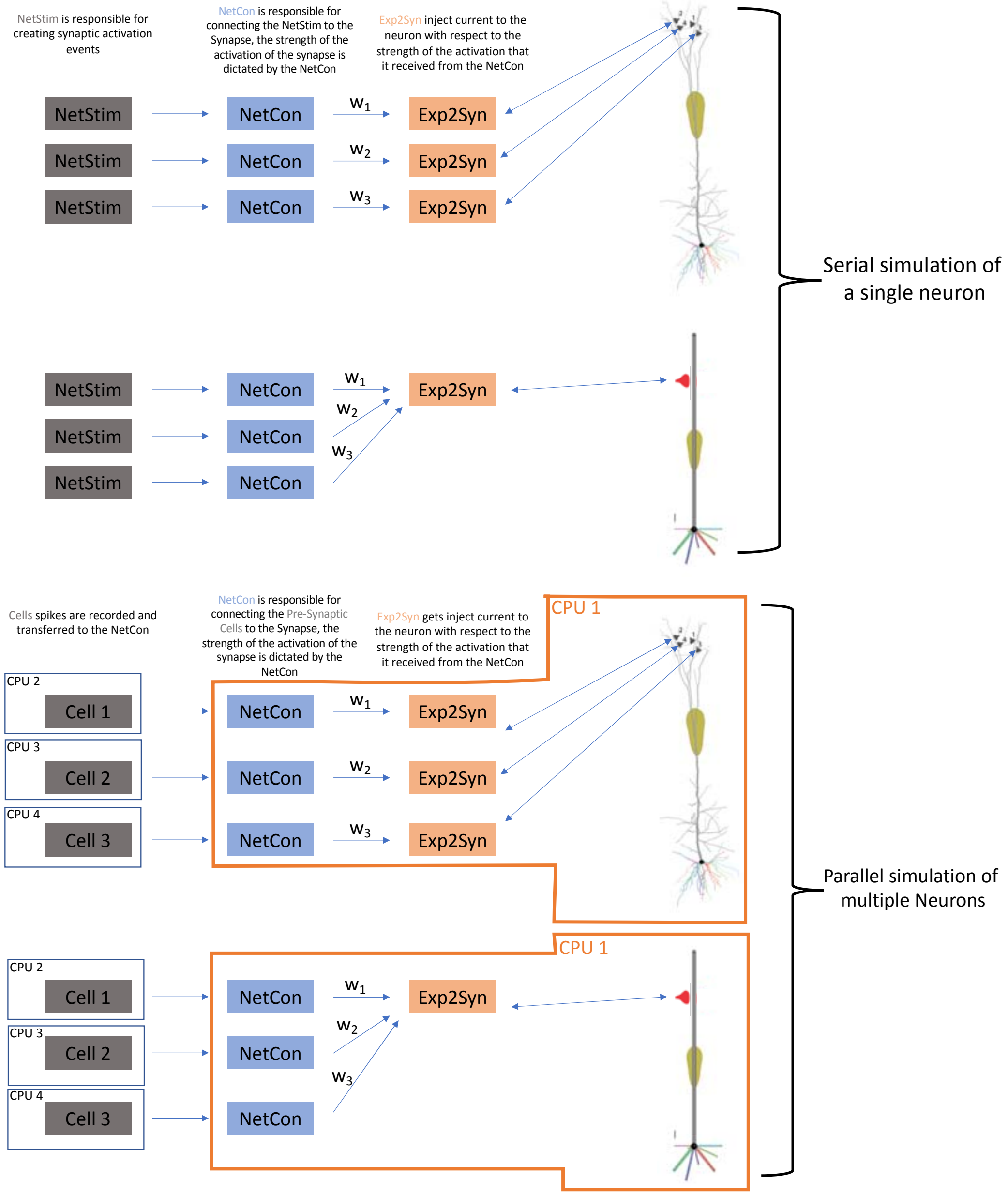

5. Page 9, top: “We also tested other spike trains similarity metrics (Figure S3) and found comparable results to those shown in Figure 3.” Should this refer to Figure 2 instead?

Corrected. Thank you

6. Page 22, third paragraph: “All synapses with a similar |Z_(0,x) (omega)| value…” – what does “similar” mean? Are they similar within some tolerance, or were they somehow binned?

We thank the referee for this comment and we now made this point clear at the **Method** section as follows:

In order to map synapses from the detailed model to the reduced one we computed, for each synapse at location j in the detailed model, $|Z_{0,j}(\omega)|$ and then mapped this synapse to the respective location y in the reduced model, such that $|Z_{0,y}(\omega)| = |Z_{0,j}(\omega)|$. This reduced model is then compartmentalized into segments (typically with spatial resolution of 0.1λ, see **Fig. 3b**). We then merged all synapses with identical kinetics and reversal potential, that are mapped to a particular segment, onto a single point process object in NEURON (**Fig. 1**, step B).

7. References: Ref. 45 is a duplication of Ref. 30; Ref. 56 is a duplication of Ref. 17.
Corrected. Thank you

Reviewer #2 (Remarks to the Author):

This study reports a systematic way to reduce complicated neuronal models. This approach is extremely interesting in principle to many modelers, as the computation time for detailed compartmental neuronal models is difficult to reduce even through parallelization.
While I am entirely in favor of the objectives, I feel that the approach needs to be better characterized, and there are some use cases that should be better explored and reported.

In my reading of the paper, I considered four specific challenges for the proposed method:
1. How does the response look at the soma?
Relevant for I/O relationships.
2. How does the local dendritic response look?
Relevant for such processes as dendritic plasticity, local summation and AP generation
3. How is the coupling with the soma?
Dendritic APs -> somatic responses.
BPAPs
4. How does intracellular chemical signaling work if the neuron is subdivided with this approach?
Relevant for local plasticity, synaptic tagging, and structural plasticity processes.

In order:

1. How does the response look at the soma?
This is pretty good. The authors did look at random input throughout the cell. However, I would like to see two additional tests:
1.1: an EI balance test, considering whether local EI balance gives the same result in the reduced model.

We thank the referee for this challenging test. We are not 100% sure that we understand what the referee expects regarding the EI balance test.

We note the following: (i). In the classical “EI balance test” (in “point” neurons (Landau et al., 2016)), the CV of the output spikes is close to 1. (ii) In our detailed model we distributed E and I synapses randomly over the dendritic surface. Thus, typically, individual dendritic branches receive the same proportion of E and I synapses (“local anatomical EI balance”).

Following the referee request, we next examined whether the CV of the output spikes in the detailed model is similar to that of the reduced model for a variety of output spike frequencies. We increased the firing rate of E (starting at 2Hz) and I synapses (starting at 4Hz) by the same factor and examined the CV of the output spikes. We found that, up to output frequency of 5 Hz, both models (detailed and reduced) have CV close to one. For higher output frequencies, the respective CVs were reduced, but it remained similar in both models (see Figure below, reduced model in red and detailed model in black). We note that, to the best of our knowledge, there is no systematic study of a “balanced EI state” in detailed (multi-compartmental) neuron models. We consider exploring this point in the future – thanks to the refree.

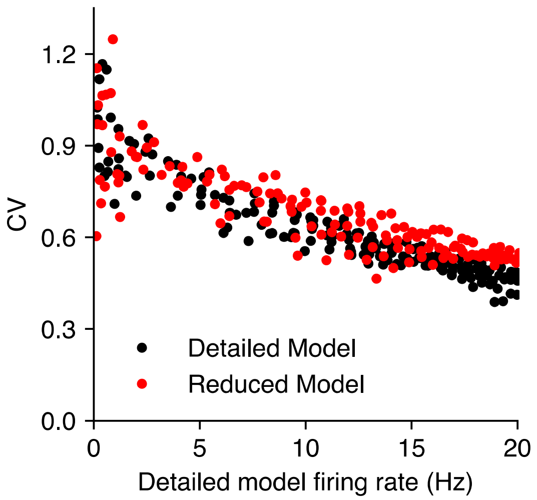

1.2: Rhythmic activity imposed by periodic inhibitory input on top of steady random background input. Does the somatic firing and impedance behave the same in full vs. reduced models?

See answer to reviewer 1, comment 2

2. How does the local dendritic response look?
2.1 This was tested for Rall's and Hausser's directionality detection. It is good to see that it works for these cases.

We thank the referee. Indeed, it was rewarding to see the similarity between the two models for directional-selective stimulation

2.2 I would like to see it tested for local (dendritic) spiking. Could the authors deliver random input with a certain distribution on the synapses, and report on triggering of local dendritic spikes?

We assume that the referee means local fast dendritic Na^{+}-spikes. In our model we do not have Na^{+}-spikes. We do have more global Ca^{2+}-spike (for which we have already tested for in comparing the detailed and the reduced model).

We have already discussed previously similar limitation for local dendritic NMDA spikes (see also Figure 2**g**). We now added the sentence below to the **Discussion** (Parag. 4) where the limitations of *Neuron_Reduced* are discussed.

Indeed, if there were highly local dendritic Na^{+} spikes (as in (Smith et al., 2013)) then *Neuron_Reduce* will not capture them, as this local dendritic spike will be averaged-out in the respective lumped cable.

2.3 The authors briefly report differences with local potentials, it would be good to have this summarized and charted for the different cases of a Ca spike on an apical dendrite, and for different frequencies of local dendritic synaptic input.

See response to the referee comment 2.4 below.

2.4 I would like to see what happens to dendritic potentials for random cell-wide input. Figs 2 and 6 don't really show this.

We thank the referee and agree that we should have shown the dendritic voltage as well. We note that, clearly, the local dendritic voltage for particular branches in the full model can not be identical to the voltage in its mapped-location in the reduced model (as the latter “integrates” voltage from different dendritic branches). In the new Figure 4 in main text (see figure below) we show the voltage traces in individual branches that are all mapped to one compartment in the reduced model. We also added the following text:

In **Figure 4** we compared the dendritic voltage in the detailed and in the respective location in the reduced model. We found that: (i) the voltage transients could differ significantly in dendritic branches that are all mapped to the same compartment in the reduced model (e.g., compare the grey traces in the yellow compartments in **Fig. 4b**). (ii) the average voltage trace in these different dendritic branches (black trace in **Fig. 4b**) is similar to the voltage in the respective compartment in the reduced model (red trace in **Fig. 4b**). The implications of the latter finding for capturing highly nonlinear local dendritic events is elaborated in the **Discussion**.

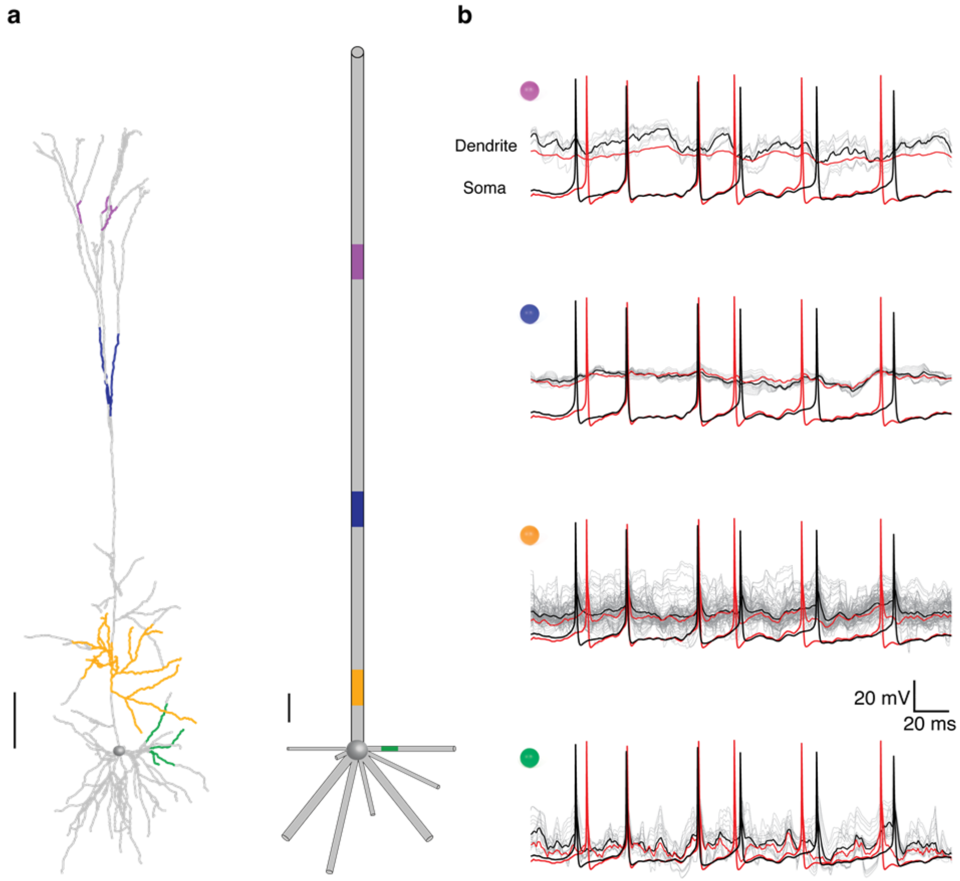

2.5 As the authors have themselves discussed in previous work, an interesting proposed computation in the cell is to have geometrically defined local inhibition, such as shunt inhibition, on one or other side of a dendritic branch. How does this carry over to the reduced model?

We assume that the reviewer refers to both our Figure 6 and Supp. 12 in (Gidon and Segev, 2012) and more recently on (Doron et al., 2018; the “off pass” versus “on path” effect of inhibition on dampening the nonlinear “hot spot”).

We note that when this phenomenon occurs locally on small dendritic branch (as depicted in (Doron et al., 2018)) this small spatial resolution is not preserved in the reduced model (namely, distal and proximal inhibition on such small branch will be mapped to same compartment in the reduced model). However, when looking at a more course spatial resolution (e.g., dampening the more spatially distributed Ca^{2+} "hotspot" via distal versus proximal inhibition) then the "off pass" versus "on path" effect of inhibition is preserved in *Neuron_Reduce* as well (see Figure below for the referee).

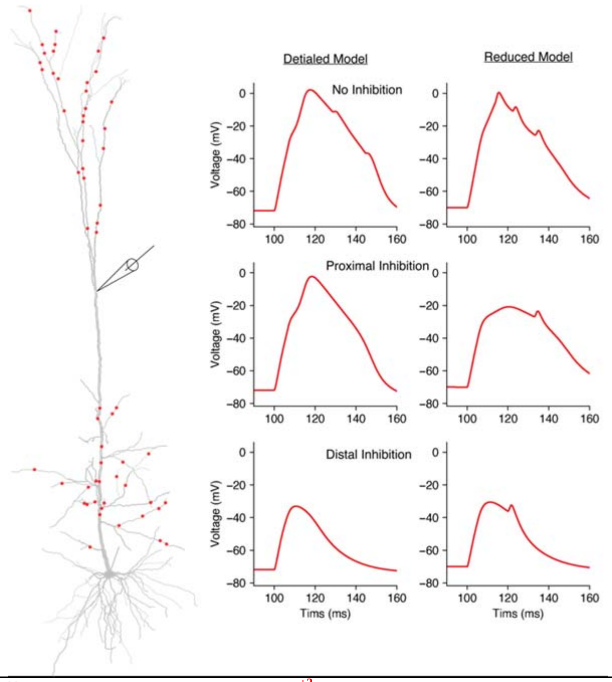

Effect of "on path" versus "off path" inhibition on dendritic Ca^{+2} spike in the detailed versus the reduced model. Ca^{+2} spike was generated by depolarizing current pulse at the "hot" apical nexus in both models. Inhibitory synapses (red circles) where distributed either "off path" (distally) or "on path" (proximal) to this "hot" region in the detailed (as in (Gidon and Segev, 2012) Figure 6 and Supp. Figure 12). These synapses where then mapped to their respective loci in the reduced model. One can see that in both models, the distal inhibition is more potent in dampening the Ca^{+2} spike.

2.6 What would happen if there were dendro-dendritic or gap-junction input to dendrites? Does this reduce cleanly or poorly?

This is an interesting expansion of our paper but it falls well into our interest in gap-junctions (Amsalem et al., 2016). In order to respond to the referee's question, we simulated two passives L2/3 basket cells connected to each other via one dendritic gap junction. The dendritic locations of the gap junction (in both cells) was selected randomly, and its conductance was either 0.1 nS or 0.2 nS. We injected a step current at the soma of cell #1 and calculated the coupling coefficient (CC) between the two cells ($\Delta V_2/\Delta V_1$). We then simplified the two cell models using *Neuron_Reduce* and mapped the locations of the gap junction so to the respective locations in the reduced models that preserve the transfer resistance from the gap junction to the soma. The resulting coupling coefficient in the two models are presented below. As can be seen in the figures below, the matching between the CC in the detailed versus the reduced models is very good.

We now added the following sentences in the revised **Discussion**.

We have also examined *Neuron_Reduce* for the case of a gap junction connecting dendrites of two L2/3 large basket cells. As *Neuron_Reduce* preserves the transfer resistance from the location of the synapses (in this case the gap junction) to the soma and *vice versa,* one expects that the coupling coefficient between the two connected cells will be preserved in the reduced models, after mapping the gap junction to its appropriate location in the reduced model. This is indeed what we have found (not shown).

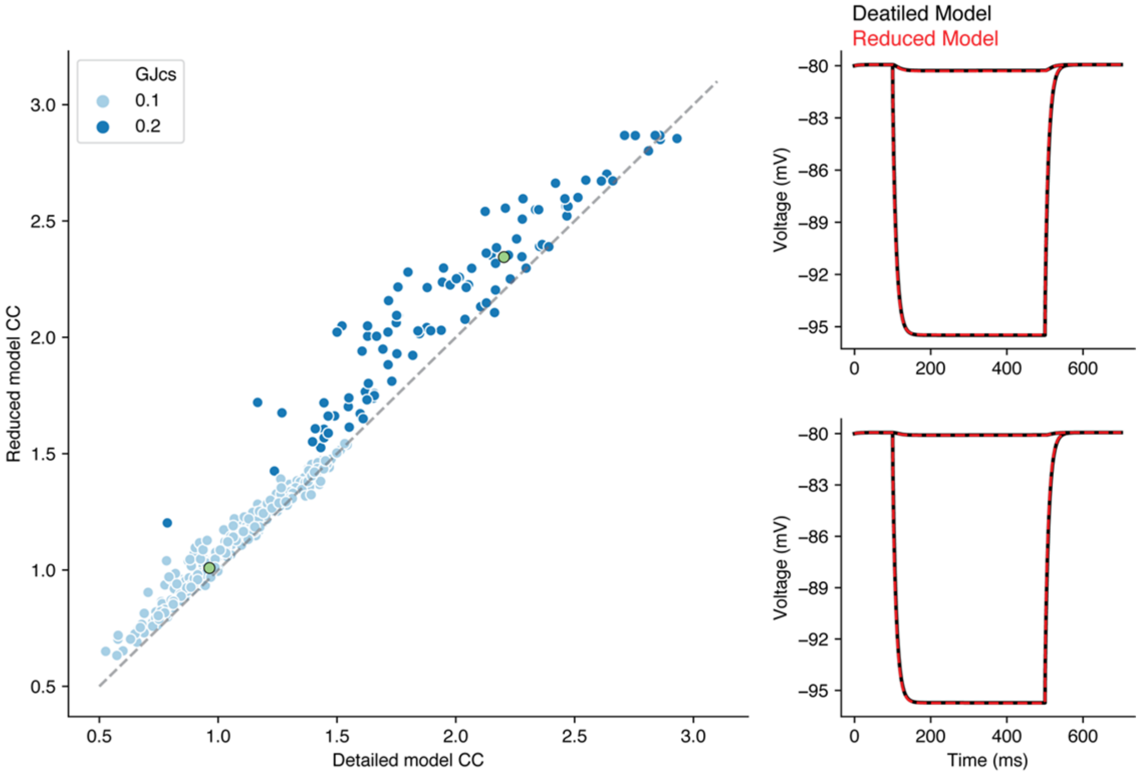

Coupling coefficient, CC, in the reduced versus the detailed models of two L2/3 large basket cells connected via a gap junction (GJ), for a variety dendritic location GJs. GJ conductance was either 0.1 nS (light blue dots) or 0.2 nS (dark blue). At right two specific cases (marked by greed circles at left) are shown.

3. How is the bidirectional coupling with the soma?
3.1 Dendritic APs -> somatic potentials.
This was done for a single dendritic Ca spike (Figure 4) but not for other forms of local dendritic activity.

We responded to this question in our response to Q 2.2 by this referee.

3.2 BPAPs
Tested. This looks reasonable.
Thanks

4. Can this mapping preserve intracellular chemical as well as electrical signaling, in local dendritic domains?

4.1 I understand that the authors have focused on electrical properties, but I would like to see at least a considered discussion on how model reduction affects this aspect of cellular signaling. For example, collapsing all synapses of a dendrite into a single synapse would seem to completely eliminate all possibility of synaptic tagging or biochemical sequence computation, not to mention reducing potential network memory capacity by orders of magnitude.

We thank the referee for this important comment. We note that collapsing the synapses to a single point process does not hinder the possibility of “synaptic tagging”. We refer the reviewer to the schematic drawing in answer to reviewer #1 minor issue 4. As presented in the schematics, when the synapses are collapsed to one Point-Process, the original NetCon for each synapse is preserved. Therefore, it retains its individuality. This implies that possibility of synaptic tagging is preserved. For example, in answer 6 to reviewer #2, the STDP per original synapse was implemented on the NetCons. Indeed, if the timing of the modeled output spikes was identical in the detailed and the reduced models, then the weight of each NetCon (of each synapse) in the detailed model and its respective NetCon (synapse) in the reduced model will change in exactly the same way (see below).

4.2 There is considerable evidence for local dendritic CICR (e.g., Plotkin's work). How could this map? I would like to see simulations.

We thank the referee for this suggested extension. However, CICR simulations is much beyond the scope of the present work, which really focuses on “Electrical reduction” of the detailed model.

Beyond these specific challenges, and as a further test of the approach, here are a couple of suggestions:

5. Introduce a reduced cell as a replacement for a detailed cell in a regular Blue-brain type cortical simulation. Ask how its spiking output compares with the output of the original cell.

We now did simulate a Blue Brain input to a single (detailed and reduced) cell – see comment 2 of Reviewer #1. Indeed, we plan in the near future to replace all neurons in the BB simulation with their respective reduced models.

6. In the same cell, ask if the synapses obeyed STDP rules, would they change in the same way? The authors should compare with original detailed model.

We thank the referee for suggesting this interesting benchmark. In principles, since the somatic spike trains are very similar in both full and reduced models, and the identity (timing) of individual synapses is retained in the reduced model (the original NetCon for each synapse is preserved) – then one expects similar STDP trajectories in both cases. Following the referee question, we implemented a full adaptive STDP protocol for all 8,000 excitatory synapses as in (Gidon and Segev, 2009; Song et al., 2000) for a 100 sec. simulation time. All synapses started with a peak conductance of 0.4 nS and a cap-conductance of 0nS - 0.8 nS. The Figure below shows the conductance value at the end of the simulation for all excitatory synapse in the simplified vs. the detailed model. This comparison shows that, following STDP, the change in conductance of the respective synapses is correlated between the two models (Person correlation; p = 0 for the left case). For comparison, we show in the right frame below the case where for each synapse in the detailed model we randomly selected a synapse in the reduced model. Now p = 0.872.

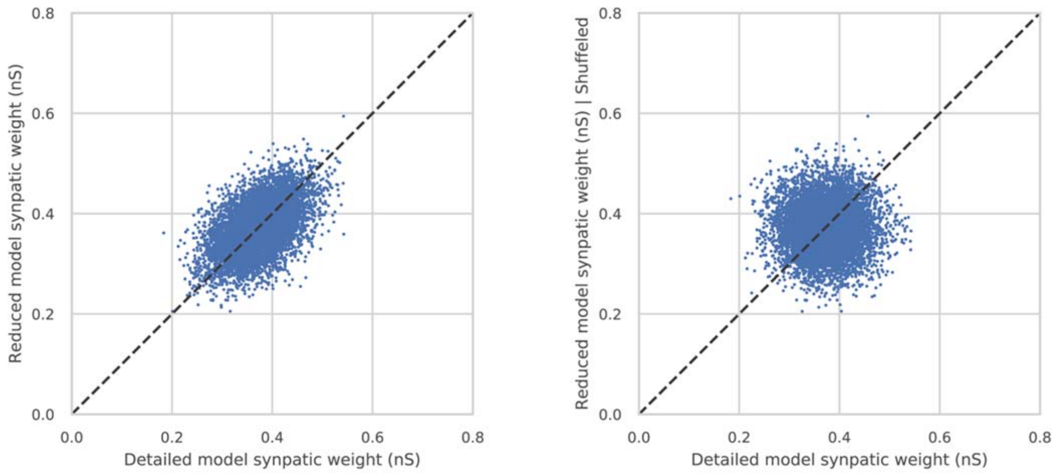

7. I also have a technical concern about the stated speedup with respect to

synapses. In very large network simulations, much of the computation time is actually spent on synaptic transmission. This is even more so in the case of plastic synapses.
While it may be true that this method gives a 10-100 fold speedup for the cell model alone, it wasn't clear to me how the collapsed synapses were handled and how this scaled.

See answer 4 in minor issues of reviewer #1 and our response to point 7.2 below

7.1 For example, if you have 100 inputs coming onto a single synaptic point process on a compartment, are the weights still changing independently?

Yes, they are, see schematics in answer 4 in minor issues of reviewer #1

7.2 I would like to see a test of this proposed speedup for a network. My impression is that more than half the computation time may be spent in transmission and synaptic rules, so all that this method could do would be to improve the runtime by 50% or so, and possibly much less. This should be explicitly measured for simple and for plastic synapses. My sense is that the shared synaptic point process may not be the major term in the computational cost for large numbers of synapses.

We note the following:

1. When benchmarking the Blue Brain simulations, we found that spike transmission between CPUs takes ~ %5 of the total simulation time (Kumbhar et al., 2019). This implies that, theoretically, the speed-up is bounded by a factor of 20X when simplifying a full network using *Neuron_Reduce* (or, for that matter, any other single cell reduction method). We agree that the reduction in neuron computational will make spike communication the dominant part of the overall execution. In this case, there are different ways spike communication can be implemented to improve performance. For example, (Hines et al., 2011) have suggested a new methods (DCMF_Multicast) which could be used with newer network support to handle spike transmission more efficiently than what is used today in the NEURON simulator. Also, as in NEST (Fernandez-Musoles et al., 2019), neurons can be distributed across CPUs such that synaptically connected cells are mapped to nearby CPUs; this reduces the communication time. Benchmarking *Neuron_Reduce* with the above spike-exchange methods is highly time consuming and not within the scope of the present study. This will be tested in the future when *Neuron_Reduce* will be incorporated in the somatosensory microcircuit model of Blue Brain simulations.

2. For plastic synapses with simple STDP rule (as used in the simulations depicted in the Figure above) – the updating rule for the synaptic weight is straightforward and parallelizable, therefore it does not require much computation time. But this clearly depends on the complexity of the STDP rule and requires specific benchmarking per specific rule.

Overall, I think that the method is potentially very interesting. However, for the stated goal of the authors (to reduce highly detailed cell models for use in large network simulations) I feel that the current analysis doesn't go far enough. Specifically, there are a number of likely dendritic input scenarios in which I suspect the approach may provide degraded accuracy. These should be well characterized and systematically reported (e.g., in a table) for readers to be better able to decide when to use such a model reduction approach. I also have a technical concern about whether the reported speedup really holds in a network full of plastic synapses.

We believe that we responded to (almost all) of the referee's requests – which were indeed demanding but we feel that, consequently, the paper was significantly improved. We therefore thank the referee for his fruitful comments.

References for the referee

Amsalem, O., Van Geit, W., Muller, E., Markram, H., and Segev, I. (2016). From Neuron Biophysics to Orientation Selectivity in Electrically Coupled Networks of Neocortical L2/3 Large Basket Cells. *Cereb. Cortex* 26, 3655–3668. doi:10.1093/cercor/bhw166.

Doron, M., Chindemi, G., Muller, E., Markram, H., and Segev, I. (2018). Shaping Dendritic

NMDA Spikes by Timed Synaptic Inhibition: Implications for Branch-Specific Synaptic Plasticity and I/O Properties of Cortical Neurons. *SSRN Electron. J.* doi:10.2139/ssrn.3155777.
Fernandez-Musoles, C., Coca, D., and Richmond, P. (2019). Communication Sparsity in Distributed Spiking Neural Network Simulations to Improve Scalability. *Front. Neuroinform.* doi:10.3389/fninf.2019.00019.
Gidon, A., and Segev, I. (2009). Spike-timing-dependent synaptic plasticity and synaptic democracy in dendrites. *J. Neurophysiol.* 101, 3226–34. doi:10.1152/jn.91349.2008.
Gidon, A., and Segev, I. (2012). Principles Governing the Operation of Synaptic Inhibition in Dendrites. *Neuron*. doi:10.1016/j.neuron.2012.05.015.
Hines, M., Kumar, S., and Schürmann, F. (2011). Comparison of neuronal spike exchange methods on a Blue Gene/P supercomputer. *Front. Comput. Neurosci.* 5, 49. doi:10.3389/fncom.2011.00049.
Kumbhar, P., Awile, O., Keegan, L., Alonso, J. B., King, J., Hines, M., et al. (2019). An optimizing multi-platform source-to-source compiler framework for the NEURON MODeling Language. Available at: http://arxiv.org/abs/1905.02241 [Accessed August 10, 2019].
Landau, I. D., Egger, R., Dercksen, V. J., Oberlaender, M., and Sompolinsky, H. (2016). The Impact of Structural Heterogeneity on Excitation-Inhibition Balance in Cortical Networks. *Neuron*. doi:10.1016/j.neuron.2016.10.027.
Markram, H., Muller, E., Ramaswamy, S., Reimann, M. W., Abdellah, M., Sanchez, C. A., et al. (2015). Reconstruction and Simulation of Neocortical Microcircuitry. *Cell* 163, 456–92. doi:10.1016/j.cell.2015.09.029.
Smith, S. L., Smith, I. T., Branco, T., and Häusser, M. (2013). Dendritic spikes enhance stimulus selectivity in cortical neurons in vivo. *Nature* 503, 115–120. doi:10.1038/nature12600.
Song, S., Miller, K. D., and Abbott, L. F. (2000). Competitive Hebbian learning through spike-timing-dependent synaptic plasticity. *Nat. Neurosci.* doi:10.1038/78829.

Reviewers' comments:

Reviewer #1 (Remarks to the Author):

The authors have done much work to address the reviewers' comments. I appreciate the effort it took. The manuscript has been improved substantially.

However, I had hoped to see more in these revisions. My two major concerns in the first review were that (1) the study showed performance of Neuron_Reduce on only N=8 neurons and (2) did not show its performance on realistic input that neurons would receive in the context of an active cortical network. The authors addressed both points, but for (1) they only increased N from 8 to 13 and for (2) they only did the analysis for N=1 neuron. Regarding point (1), one suggestion was to try Neuron_Reduce on non-mammalian neurons, but the authors stated that they did not find good non-mammalian cell models; however, a quick search of ModelDB shows plenty of NEURON models for non-mammalian species, such as turtle, owl, fly, leech, crab and others. The authors also did not follow the suggestions to demonstrate additional measures beyond SPIKE-synchronization for multiple neurons or to compare the Neuron_Reduce performance with simpler point-neuron models.

While all of these could be useful additions, I appreciate the fact that the authors have already done a lot of work for this manuscript. I really like the Neuron_Reduce method and the fact that it is publicly available on GitHub and I believe that it may be useful for many scientists. Perhaps one does not need to follow all the suggestions above. But I do think that demonstrating the method's performance on a sufficiently large N of neurons would be very useful to judge its applicability to real biological problems. With N=13, all from different neuron types, we do not understand the variability of the method's performance within type and across types.

As scientists, we would like to know the statistics of the method's performance. Since the method is easy to use and computationally inexpensive, and the authors have in their possession a large library of detailed neuronal models, why not show the performance of the method on this library? Do that for the most prominent cell types in the library (say, excitatory neurons from each cortical layer and several most common inhibitory types), perhaps N=10 neurons for each type, then show box plots of SPIKE-synchronization in each frequency bin (like in Fig. 6g) for each type. This would be very helpful and convincing.

Given that the method is fast and easy to use, I would consider this a minor revision that could substantially improve the paper's impact.

Reviewer #2 (Remarks to the Author):

The authors have addressed the reviewer comments very thoroughly.
There was a bit of a misunderstanding regarding my question about the EI balance, in which I had asked about local balancing (within a small dendritic section) vs cell-wide balancing. Nevertheless, I do not regard this as a major issue and I feel that the other analyses give an idea of how well this reduction would work.
I compliment them on making this method for systematic model reduction with a broad and convincing range of benchmarks for the accuracy of the reduced model.

Reviewer #1 (Remarks to the Author):

The authors have done much work to address the reviewers' comments. I appreciate the effort it took. The manuscript has been improved substantially.

However, I had hoped to see more in these revisions. My two major concerns in the first review were that (1) the study showed performance of Neuron_Reduce on only N=8 neurons and (2) did not show its performance on realistic input that neurons would receive in the context of an active cortical network. The authors addressed both points, but for (1) they only increased N from 8 to 13 and for (2) they only did the analysis for N=1 neuron. Regarding point (1), one suggestion was to try Neuron_Reduce on non-mammalian neurons, but the authors stated that they did not find good non-mammalian cell models; however, a quick search of ModelDB shows plenty of NEURON models for non-mammalian species, such as turtle, owl, fly, leech, crab and others. The authors also did not follow the suggestions to demonstrate additional measures beyond SPIKE-synchronization for multiple neurons or to compare the Neuron_Reduce performance with simpler point-neuron models.

While all of these could be useful additions, I appreciate the fact that the authors have already done a lot of work for this manuscript. I really like the Neuron_Reduce method and the fact that it is publicly available on GitHub and I believe that it may be useful for many scientists. Perhaps one does not need to follow all the suggestions above. But I do think that demonstrating the method's performance on a sufficiently large N of neurons would be very useful to judge its applicability to real biological problems. With N=13, all from different neuron types, we do not understand the variability of the method's performance within type and across types.

As scientists, we would like to know the statistics of the method's performance. Since the method is easy to use and computationally inexpensive, and the authors have in their possession a large library of detailed neuronal models, why not show the performance of the method on this library? Do that for the most prominent cell types in the library (say, excitatory neurons from each cortical layer and several most common inhibitory types), perhaps N=10 neurons for each type, then show box plots of SPIKE-synchronization in each frequency bin (like in Fig. 6g) for each type. This would be very helpful and convincing.

Given that the method is fast and easy to use, I would consider this a minor revision that could substantially improve the paper's impact.

We thank the referee for his acknowledgment of the improvement of the paper and his appreciation of the work that has been done.

In response to the referee comments

1. We have re-looked again at the available database for non-mammalian neurons. Unfortunately, not all models have a 3D morphology, and those that do have either lack systematic modeling of their excitability properties or the morphology is already extremely simple and does not require further reduction.

2. However, we did take the 2nd comment of the review to its full extent. As suggested by the referee, we selected 8 morphological types (2 from each layer 2-6, taken from the Blue Brain dataset) and selected 11 cells for each cell type (88 modeled cells in total). For each neuron, we searched for the correct excitatory synaptic input that will result, in its respective detailed model, with an output firing rate that covered the range between 0-20 Hz.

   The pooled results are presented in the revised manuscript in a new **Supplementary Figure 6** and are referred to in the appropriate locations in the revised main text (see revised manuscript). As can be seen in this Figure, the Spike-synchronization is within the range of that found in **Figure 7**. Also, these simulations show how the variance between the different cell-types is larger than the variance between the cells within each type.

Simulation of 88 neurons actually required extensive work and simulation time. Yet, it did provide an insightful addition for the paper in particular regarding the variability in the quality of *Neuron_Reduce* performance for both within-cell-type and between-cell-types. **We thank the reviewer for his suggestion**.

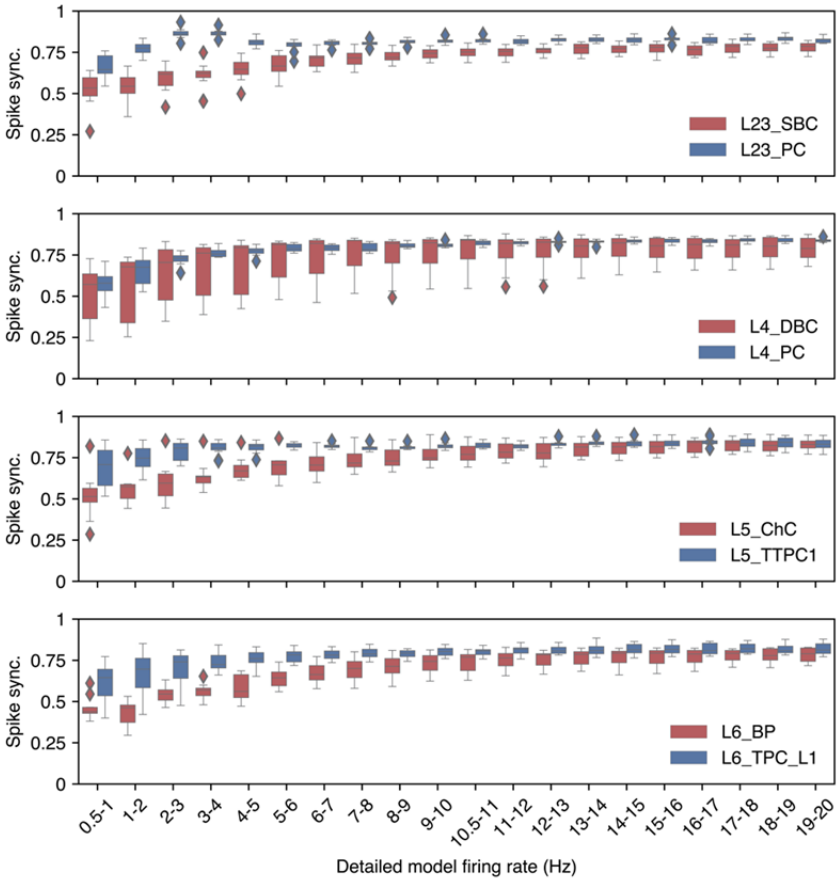

Supplementary Figure 6. The accuracy of *Neuron_Reduce* on a large sample of neocortical neurons. Boxplot of Spike-synchronization measured on 8 morphological types from the Blue Brain database[5,6]. 11 models were sampled for each type (88 cells in total), and simulations were executed for 30 seconds for each output frequency (x-axis). Each excitatory neuron model received 10,000 synapses, and each inhibitory neuron received 3,000 synapses. Synaptic input for the excitatory neurons was as for tufted PC (L6) shown in **Supplementary Table 2** (5th row). Synaptic input for inhibitory neurons was as for the L5 Martinotti cell shown

in **Supplementary Table 2** (9th row). The number of compartments in the reduced model was set to 10% of the number of compartments in the detailed model when spatial discretization, ΔX, per compartment of 0.1λ resulted in a total number of compartments that is lower than 10% of the number of compartments in the detailed model (See **Supplementary Table 1**). SBC – Small Basket Cell; DBC – Double Bouquet Cell; ChC – Chandelier Cell; BP – Bipolar Cell; PC – Pyramidal Cell; TTPC1 – Thick-tufted Pyramidal Cell with a late bifurcating apical tuft; TPC_L1 – Tufted Pyramidal Cell with apical dendrites terminating in layer 1. Box plots bounds spans from 25 to 75% percentile, center line represents median, and whiskers extend to $\pm$ 1.5 interquartile range.

Reviewer #2 (Remarks to the Author):

The authors have addressed the reviewer comments very thoroughly.
There was a bit of a misunderstanding regarding my question about the EI balance, in which I had asked about local balancing (within a small dendritic section) vs cell-wide balancing. Nevertheless, I do not regard this as a major issue and I feel that the other analyses give an idea of how well this reduction would work. I compliment them on making this method for systematic model reduction with a broad and convincing range of benchmarks for the accuracy of the reduced model.

We thank the reviewer for his constructive input in the first round and for his appreciation of the extensive work invested in the paper.

REVIEWERS' COMMENTS:

Reviewer #1 (Remarks to the Author):

The authors added to their study the coarse-graining results for a large number of neurons. The large computational expense and amount of work that went into this are highly appreciated. I am happy to see that these new result strongly support the authors' conclusions and illustrate applicability of the method.

All my concerns have been addressed, and I recommend publication. I congratulate the authors on the great study, which will be of high interest to the community.

REVIEWERS' COMMENTS:

Reviewer #1 (Remarks to the Author):

The authors added to their study the coarse-graining results for a large number of neurons. The large computational expense and amount of work that went into this are highly appreciated. I am happy to see that these new result strongly support the authors' conclusions and illustrate applicability of the method.

All my concerns have been addressed, and I recommend publication. I congratulate the authors on the great study, which will be of high interest to the community.

We thank the reviewers and editors for their constructive input.
